# The Influence of Sex, Age, and Race on Coronary Artery Disease: A Narrative Review

**DOI:** 10.7759/cureus.47799

**Published:** 2023-10-27

**Authors:** Célia C Lima Dos Santos, Arshveer S Matharoo, Emilio Pinzón Cueva, Uzma Amin, Aida A Perez Ramos, Navpreet K Mann, Sara Maheen, Jyothsna Butchireddy, Vaibhavkumar B Falki, Abeeha Itrat, Nithyalakshmi Rajkumar, Muhammad Zia ul Haq

**Affiliations:** 1 Cardiology, Eberhard Karl University of Tübingen, Tübingen, DEU; 2 Cardiology, Avalon University School of Medicine, Willemstad, CUW; 3 Cardiology, Anáhuac University, Mexico City, MEX; 4 Pathology, Rawalpindi Medical University, Rawalpindi, PAK; 5 Cardiology, Universidad Central de Venezuela, Caracas, VEN; 6 Cardiology, Government Medical College and Rajindra Hospital, Patiala, IND; 7 General Medicine, Odessa National Medical University, Odessa, UKR; 8 Cardiology, Government Medical College, Omandurar Government Estate, Chennai, IND; 9 Cardiology, Corewell Health University Hospital, Michigan, USA; 10 Cardiology, Lutheran General Hospital, Illinois, USA; 11 Cardiology, Kilpauk Medical College, Chennai, IND; 12 Epidemiology and Public Health, Emory University Rollins School of Public Health, Atlanta, USA; 13 Noncommunicable Diseases and Mental Health, World Health Organization, Cairo, EGY

**Keywords:** ethnicity, race, age, sex, artery disease

## Abstract

Cardiovascular disease has remained one of the leading causes of mortality in the world. The basic pathophysiology of coronary artery disease (CAD) is a reduction of the blood flow in coronary vessels, leading to restricted blood flow to the heart muscle. Both modifiable and non-modifiable risk factors contribute to its multifactorial etiology. The clinical presentation ranges from asymptomatic to typical symptoms like chest pain, shortness of breath, and left arm or jaw pain. The purpose of this review is to investigate and analyze the variation of CAD depending on the biological sex, age, race, or ethnicity and how it might differ in the studied population while comparing the symptoms and prognosis of CAD.

For this research, PubMed’s database was used. A total of 926 articles were selected using pre-determined inclusion and exclusion criteria, with 74 articles eligible to be included in the narrative review. Studies were selected from the general population of patients with CAD, regardless of their severity, stage of diagnosis, and treatment plan. The scale for the assessment of non-systematic review articles (SANRA) was used to assess the quality of the study.

As humans age, the incidence of CAD increases, and people over 75 are more likely to have multiple-vessel CAD. It has been observed that South Asians have the highest rate of CAD at 24%, while the White population has the lowest at 8%. The prevalence of CAD also depends on race, with the White population having the lowest rate at 3.2%, followed by Hispanics at 5%, Black women at 5.2%, and Black men at 5.7%. Younger Black women tend to have more chest pain. Men with CAD commonly experience chest pain, and women are more likely to present with atypical symptoms. Modifiable risk factors such as smoking and alcoholism are more commonly observed in young men than in young women.

Coronary artery disease in the elderly, female, minority, and Black patients is associated with a higher mortality rate. Acknowledging the prevalence of certain risk factors, signs, results, and responses to treatment in certain socio-demographic groups, as well as the provision and accessibility of diagnosis and treatment, would lead to a better outcome for all individuals. The impact of this shift can range from an earlier diagnosis of CAD to a faster and more customized treatment plan tailored to each patient's individual requirements.

## Introduction and background

Cardiovascular disease (CVD), including ischemic heart disease, has been a burden of disease in both men and women [1]. Its pathophysiology involves a reduction in coronary blood flow to the myocardium, which causes myocardial ischemia. This condition is mainly due to atherosclerosis, a mild inflammatory condition of the internal layer (intima) of medium-sized arteries in which macrophages engulf low-density lipoproteins and form what are called foam cells, which accumulate and contribute to the formation of atherosclerotic plaques that reduce the diameter of medium-sized arteries, therefore restricting the flow of blood. However, other non-obstructive pathophysiological mechanisms (e.g., coronary vasospasm) also have an important role in the progression of coronary artery disease (CAD) [2].

Recognizing the risk factors involved with the development of CAD is an essential part of the diagnosis. Among the non-modifiable risk factors are age, race, ethnicity, sex, and family history. Modifiable risk factors include elevated cholesterol levels, high blood pressure, tobacco use disorder, physical inactivity, a high body mass index, diabetes mellitus, and a poor diet, among others [3,4]. 

Typical symptoms of CAD include central oppressive chest pain, jaw and left arm pain, diaphoresis, shortness of breath, nausea, and vomiting [5-10]. Atypical symptoms include fatigue, epigastric pain, dull or burning pain, palpitations, lightheadedness, neck pain, indigestion, shoulder pain, right arm pain, back pain, dizziness, or even syncope. Atypical symptoms are more prevalent in women and diabetic patients, and these symptoms are usually overlooked by healthcare providers [5-12]. A study suggests that labeling symptoms as 'typical' or 'atypical' should no longer be done as the presentation of CAD varies with age, sex, race, and other preexisting risk factors [8].

When diagnosing symptomatic patients with suspected CAD, it's important to conduct a thorough evaluation that includes assessing their coronary architecture and luminal constriction. One effective and accurate procedure for achieving this is coronary computed tomography angiography (CCTA), which provides a complete and noninvasive assessment of atherosclerosis. Additionally, it enables advanced plaque analysis and quantification. This test is highly reliable for the diagnosis of CAD, having a sensitivity of 96% and a specificity of 92% [5]. Another effective diagnostic method is the coronary artery calcium score (CACS), which has a sensitivity of 95% and a specificity of 66% and is an effective tool for risk stratification, particularly in women. Exercise treadmill testing, dobutamine stress echocardiography and exercise stress echocardiography tests have a lower sensitivity (<80%) when compared to the sensitivity of CCTA and CACS tests; however, their specificity is greater (>70%) than the specificity of CACS. An electrocardiogram is another routinely administered diagnostic modality to detect CAD; however, the specificity is not as high as the other tests. One essential factor in the evaluation of this condition is the identification of non-modifiable risk factors related to coronary heart disease (CHD), as they represent 63% to 80% of the total prognostic model performance [4].

Nearly half of the US population is expected to have some kind of cardiovascular disease by 2035, and according to research, the burden of CAD disease in other wealthy or developed countries is predicted to increase in the years to come, making it a global concern [1]. The purpose of this review is to investigate and analyze the variation of CAD depending on the biological sex, age, race, or ethnicity and how it might differ depending on the studied population.

We define CAD as a burden of diseases affecting coronary circulation, including stable angina, unstable angina, and acute myocardial infarction (AMI) (with or without ST-segment elevation). Coronary artery disease can be either obstructive (atherosclerotic) or non-obstructive. When obstructive CAD is mentioned, it refers to stenosis of the coronary arteries of 50% or more, which significantly impairs the blood flow to the myocardium.

Methodology

**Figure 1:**
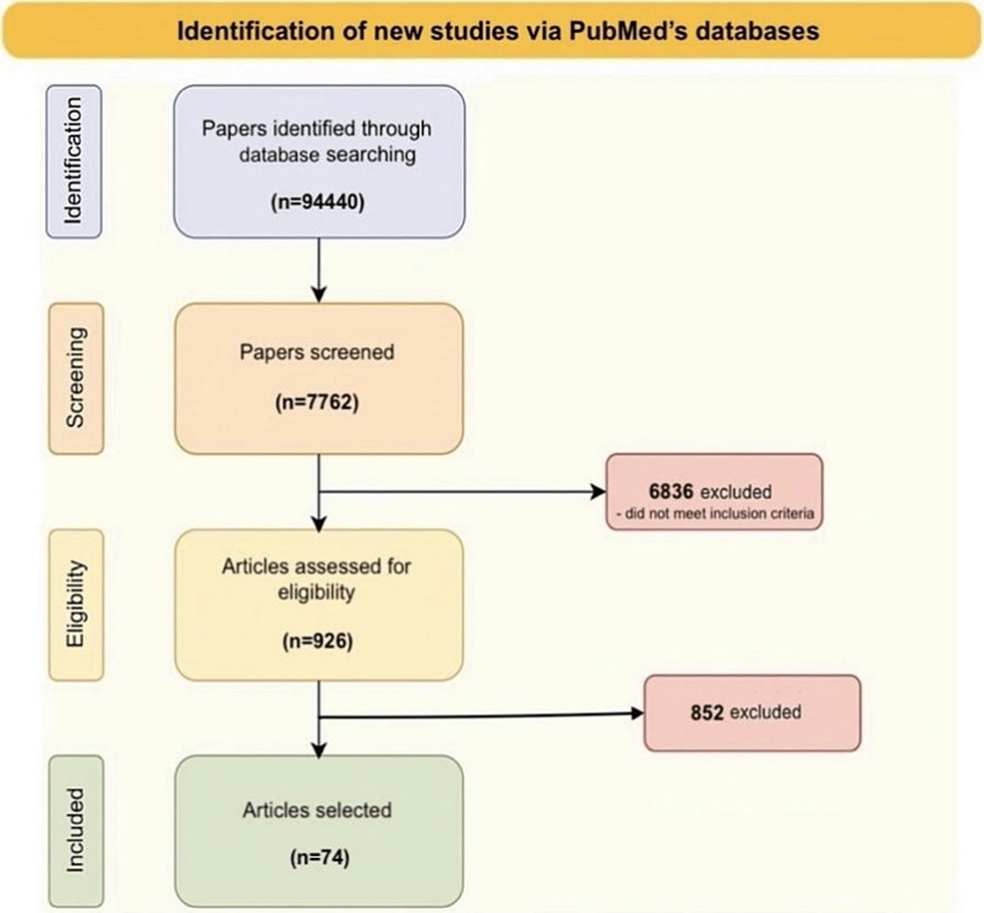
Identification of new studies via PubMed’s databases Results from the search conducted for the identification of new studies via PubMed’s database.

For this research, PubMed’s database was used, using keywords “sex” OR “gender” OR “age” OR “race” OR “ethnicity”, added with an AND to “coronary artery disease” OR “CAD” OR “myocardial infarction” OR “coronary disease” OR “acute coronary syndrome” OR “STEMI” OR “NSTEMI” OR “ischemic heart disease” OR "IHD", together using AND with “prevalence” OR “epidemiology” OR “presentation” OR “clinical features” OR “outcomes” OR "disparities" OR “risk factors”. For the initial identification stage, no filters were applied, and inclusion and exclusion criteria were not taken into consideration at this stage. The results showed a total of 94,440 articles that were initially selected in the identification stage (Figure 1).

Out of the total results, 926 articles were further selected using pre-determined inclusion and exclusion criteria that were applied to the filter features in PubMed's database. Two criteria set for this study are the study type and the date of publication of the study, so for this narrative review only original research studies published in the last 15 years (including 2008), such as observational studies (cross-sectional, case-control, and cohort studies), randomized controlled trials (RCTs), registry-based studies, systematic reviews, and meta-analyses, were included. The population criterion for this study is patients diagnosed with CAD, irrespective of severity, staging, and treatment regimen. Thus, case reports, conference abstracts, editorials, commentaries, letters, and narrative reviews (or other studies without primary data), studies involving animals, in vitro studies, and studies in which the population is not diagnosed with CAD were excluded. Also, studies with full text not available or missing data that couldn’t be obtained after contacting the study authors were not used.

The inclusion criteria also required that only studies that assessed the influence of at least one of the following demographic factors on CAD outcomes, i.e., sex, age, race, or ethnicity, could be included and that these studies should report one or more of the following: disease presentation, risk factors, disease presentation, disease progression, response to treatment, clinical outcomes (mortality, morbidity), or quality of life. Therefore, studies that do not provide information regarding the outcomes and evaluate the demographic factors specified were not selected. An important criterion was the language, so for this review, the studies selected were published in English or in other languages that have comprehensive English abstracts.

After applying the inclusion and exclusion criteria and reading the studies, 74 articles were finally eligible and selected to be included in the narrative review. The selected articles were thoroughly reviewed to understand the multi-factorial broad spectrum of CAD and its implications in present-day clinical medicine. The findings are articulated in a manner that provides a holistic picture and emphasizes the importance of various demographic variables on the clinical progression, presentations, and outcomes of CAD. The quality of the studies was evaluated using the scale for the assessment of non-systematic review articles (SANRA). Comprising six items, the quality of the article is rated from zero to two, indicating low to high quality.

## Review

Influence of sex on CAD

Prevalence and Risk of CAD in Males Versus Females

There is a hypothesis that CAD affects both men and women with a similar prevalence; however, there is still a lack of information regarding specific epidemiological differences between men and women. Several studies have approached different aspects regarding the prevalence and risk factors differences between both sexes, and some important variations are worth mentioning [9,11-22]. Many studies have proven that women present with CAD at a later age and with a greater number of comorbidities, especially high blood pressure, diabetes mellitus, renal failure, and prior congestive heart failure, whereas men have shown a higher smoking habit, being overweight, prior myocardial infarction (MI), and going through a previous percutaneous coronary intervention (PCI) [9,11,13-17,21]. 

A prospective multinational registry study concluded that both sexes show no significant difference in the rates of obstructive CAD; however, in this publication, when they analyzed CAD in general, the male sex demonstrated a higher prevalence than women (21% vs. 12%) [18]. Particularly talking about the development of the disease, women present more often with non-obstructive CAD, coronary vasospasm, takotsubo cardiomyopathy, and microvascular dysfunction than men. This same paper associated major adverse cardiovascular events with the presence of emotional alterations and stated that men had a higher risk of having psychological risk factors (anxiety, depression) than women, and consequently, men have more unfavorable CAD outcomes [19].

When it comes to diagnosis and treatment, the female sex tends to have less likelihood of receiving coronary angiography for diagnostic and therapeutic methods [12-13,15,20]. Women also showed lower chances of receiving guideline-based medical treatment like aspirin, P2Y12 inhibitors, beta-blockers, or statins in the context of CAD; however, they were more likely to receive antianginal drugs, non-steroidal anti-inflammatories, antihyperglycemic drugs, proton pump inhibitors, or thyroid replacement hormones compared to men [13,14,16,20]. A study showed that women undergoing coronary angiography had a lower prevalence of obstructive CAD findings when compared to men, reinforcing the idea of a higher risk of non-obstructive lesions [22].

Differences in CAD Presentation and Symptoms Between Sexes

Chest pain has been proven in several studies to be the main symptom presenting both in men and women with CAD, and it is more prevalent in the male sex, according to some studies [8-10]. However, two studies found the opposite, i.e., women might present with angina in more cases than men and that the duration of anginal symptoms is longer in men, while other studies demonstrate that women have higher odds of presenting with atypical symptoms (previously mentioned) and that women tend to perceive them more as muscular pain, stress, or anxiety, instead of relating it to heart pain [8-10,16,17,23-27]. A study reported that men tend to have “dull” or “burning” pain, and women have more “crushing” or “squeezing” pain [9,23]. Some studies might state that women have more epigastric pain, right arm pain, diaphoresis, or indigestion [24], while others have found it to be non-significant [8,10].

When patients seek medical attention after the onset of symptoms for myocardial ischemia, women are shown to present with a higher burden of comorbidities than men. Studies have proven that women are more likely to have a background of congestive heart failure, diabetes, hypertension, obesity, cerebrovascular accidents, chronic kidney disease or chronic lung disease, sedentarism, and depression [9,16,17,24,25]. On the other hand, men had higher records of current or previous hypercholesterolemia, smoking habit, ST-elevation myocardial infarction (STEMI), and proven coronary stenosis of >50% [9,24]. The prevalence of diabetes was equal for both sexes; however, a study showed that women had diabetes for a longer time than men before presenting with CAD [9,25]. Regarding ventricular function, women had better left ventricular function than men, who were more prone to suffer from left ventricular dysfunction. Apart from the past records or risk factors presented, women were on average three to six years older than men when presenting with CAD [10,16]. 

Even though men were more likely to suffer from chest pain, women had higher odds of having unstable angina for a longer time than men, as well as a worse quality of life [25]; however, this might be controversial as a study showed that women presented with a shorter duration of CAD compared to men (four years vs. five years, respectively) [16]. According to a study, in an acute episode, the average time from symptom onset to arrival at the hospital was longer in women than men (three hours vs. 2.8 hours, respectively) [26]. A couple of studies found that women showed more abnormal ECG findings and usually presented more with ST depression than men in an acute episode [9,17]. In laboratory values, it has been proven that women’s troponin values were lower at baseline than men's (3.9 mg/dL vs. 6.6 mg/dL on average, respectively), and this relationship was inverted in brain natriuretic peptide (BNP) values, being higher in women than men (165.7 mg/dL vs. 116.5 mg/dL on average, respectively) [17]. Studies have concluded that all these variations lead to a misdiagnosis of CAD in women as well as a delay in the treatment of acute episodes.

CAD-Specific Outcomes and Prognosis

Numerous research trials have been conducted so far to determine the differential impacts and outcomes of PCI vs. coronary artery bypass grafting (CABG) and anti-platelet therapy in segregated groups with CAD to formulate a better sex-specific approach to the treatment protocols [28-32]. A study designed to analyze and compare revascularization strategies among patients with unprotected left main disease noticed crucial differences. First, women had the worst baseline clinical status as it was older age, hyperlipidemia, diabetes, and they were smokers. Second, it was found that women presented more complications during and after PCI than male patients. However, when adjusted to specific ischemic events post-procedure, myocardium infarction rates were higher in women after PCI and were higher in men after CABG. Nevertheless, these differences were not significantly associated with the sex but with the presence of more comorbidities in the female group [28].

The worst outcomes for women after coronary revascularization are described in another publication. A systematic review and meta-analysis results showed that, compared to men, females with STEMI undergoing any therapeutic invasive procedure had an elevated risk of dying during the first 30 days (about four and a half weeks), which was attributed to the fact that women tended to wait longer to seek medical consultation after initiating the symptoms, which negatively impacted the door-to-balloon time. It is also more common to see heart failure, mechanical complications, and hemorrhagic events secondary to antithrombotic therapy and revascularization strategies in women [29]. Nonetheless, a randomized controlled double-blind, placebo-controlled research comparing continuing thienopyridine to placebo beyond 12 months following coronary artery stenting found that the risks of ischemic injury and bleeding coincided in both sexes [30].

A randomized study comparing two anti-platelet therapies called GLOBAL LEADERS showed differences that should be considered when choosing the individual therapeutic strategy after PCI. The method of reference consisted of the first 12 months of dual antiplatelet treatment (DAPT), followed by 12 months of aspirin alone. In contrast, the experimental method comprised DAPT during the first month and then 23 months (about two years) of ticagrelor monotherapy. After a year, men had a lower risk of bleeding during the ticagrelor monotherapy, while women presented a higher risk of hemorrhage and stroke events. In the case of DAPT strategies, there was no difference adjusted by sex [31].

A meta-analysis of a sex-specific phase III/IV randomized trial studied the potency of P2Y12 inhibitors. The efficacy and safety of the treatment, including prasugrel, ticagrelor, or intravenous cangrelor, were similar in both sexes, decreasing the occurrence of major adverse events such as death, cardiovascular mortality, and myocardium infarction. Implying that doctors can prescribe with confidence the powerful P2Y12 inhibitors without considering the sexual category [32].

Potential Biological and Sociocultural Factors

The pathophysiology underlying CAD has differences that need better understanding. Renin-angiotensin system (RAS) alteration is one significant factor to study because it precipitates cardiovascular damage. A study investigated the connection between six gene polymorphisms of RAS components and CAD in an Iranian population of 374 participants. After diagnosing CAD with coronary angiography, genotyping of six RAS gene polymorphisms was performed. The six genetic polymorphisms of RAS components studied were angiotensin-converting enzyme (ACE) (I/D, A-240T, A2350G), angiotensinogen (AGT) M235T, angiotensin II type 1 (AT1) receptor A1166C, and AT2 receptor C3123A, indicating that the TT genotype of ACE A-240T is a genetic risk factor for CAD in women [33]. Following the study of RAS, additional factors analyzed were myocardial blood flow and coronary flow reserve, which were measured in a different study. In 73 well-controlled type 2 diabetes patients without signs of obstructive CAD, they found that females had worse myocardial blood supply and diastolic function than men, the mechanism for which was suggested by a higher aldosterone response in women [34].

At a biomolecular level, cytochrome P450 (CYP) has been identified as a gene that may increase the risk of atherosclerotic disease. A specific variant known as CYP2C19 synthesizes molecules from arachidonic acids like epoxyeicosatrienoic acids (EETs) and hydroxyeicosatetraenoic acid, which help to maintain the health of blood vessels and the heart and have been found to predict CAD risk. The reduced ability of the CYP2C19 gene to produce EETs and other beneficial molecules is defined as poor metabolizing and may contribute to developing atherosclerotic disease, which includes CAD. A study comparing CAD patients with control subjects evaluated during medical checkups found that the variable of CYP2C19 genotype was not significantly linked with the development of CAD; however, when the studied population was stratified by sex, there was a significant difference, concluding that only for women, the CYP2C19 polymorphism was identified as a predictor of CAD risk [35].

In clinical practice, having proper tools to measure the damage and obtain a panorama of the CAD is essential to creating a therapeutic plan. A query concerning this is whether such diagnostic and therapeutic tools could differ between sexes for more accurate results. In univariable analysis, women had substantially higher levels of high-sensitivity C-reactive protein (CRP), an inflammatory mediator, than men and a significantly lower quantity of high-sensitivity troponin I (a biomarker of cardiac injury) than men. This study also investigated whether there were sex differences in the association of established biomarkers with the severity of CAD in stable patients undergoing coronary angiography using the SYNTAX score (used to quantify the complexity and severity of CAD based on its gravity) and the distribution and extension of coronary artery lesions observed on coronary angiography. Only high-sensitivity CRP and high-sensitivity troponin I biomarkers had higher SYNTAX scores and therefore were associated with more severe CAD, and posterior to this adjustment, the study did not find any significant sex variation [36].

While focusing on the manifestations accompanying the physiopathology process of CAD, it must be considered that women tend to present signs and symptoms of CHD later in life compared to men. It is believed that because hormones protect the arterial endothelium, women require more time and the accumulation of risk factors to develop atherosclerotic heart disease. In the long term, results indicate that female sex is related to a better quality of life, although when depressive symptoms occur, women lose this advantage. This association shows that emotional state, particularly in women, may be crucial for improving prognosis in patients with CHD [37].

The onset and progression of CAD involve more than just biological factors. However, it is difficult to understand how demographic characteristics could influence the pathophysiologic mechanism of CAD and needs further investigation. What is clear is the existence of differences in the development, severity, and prognosis of CAD between sexes when they are categorized by specific social and psychological attributes. Regarding this matter, women with CAD had worse mental and physical health when compared with men [38]. A different clinical trial found that young women had worse physical conditions and mental functioning than men of the same age prior to an episode of AMI. These findings suggest that targeting the context prior to the CAD event has the potential to identify high-risk female individuals early on [39].

Negative emotions have been related to the socioeconomic stratification of depressed CAD patients. A study from China showed that women with CAD were more likely to be retired or manual workers, with an average lower education and a lower socioeconomic status than men [14]. The Stepwise Psychotherapy Intervention for Reducing Risk in Coronary Artery Disease (SPIRR-CAD) trial, a multicenter study in Germany that studied patients with CAD and analyzed the social and economic status of the studied population, concluded that women with lower levels of education had less social and emotional support, while academic men had the most [40]. Following the practical implications of social status, specifically marital status, there is a meaningful influence on the severity of the condition in females; an observational, multicenter study reported that there is a significant association between divorce and severe multivessel disease, or left main disease, in women [41].

In some of the studies analyzed, there is still a contradiction in the results; some do not show prevalence differences of CAD [19], while another investigation indicates it is higher in the male group [18]. These inconsistencies demonstrate the importance of conducting further research to prove where the variation in prevalence between sexes stands, if any, and the reason for its occurrence. Another important point to consider is to clarify why female groups tend to present CAD with less compensatory vascular modification and an increased rate of during-procedure and post-PCI complications [28]. Therefore, it is essential to adjust each group's comorbidity burden, hormonal and inflammatory condition, and mental health status, as in both women and men, the strong association between mental health and social status has been demonstrated [18], emphasizing the influence of emotional support on the progression and prognosis of the disease. The summary of the findings from the referenced studies on the influence of sex as a non-modifiable risk factor on CAD is in Table 1.

**Table 1:** The impact of sex on CAD CAD: Coronary artery disease, CVD: Cardiovascular disease, CHD: Coronary heart disease, NSTEACS: Non-ST elevation acute coronary syndromes, ACS: Acute coronary syndrome, AMI: Acute myocardial infarction, CV: Cardiovascular, MI: Myocardial infarction, DM: Diabetes mellitus, HT: Hypertension, CHF: Congestive heart failure, CKD: Chronic kidney disease, PCI: Percutaneous coronary intervention, NSTEMI: Non-ST-elevation myocardial infarction, GDMT: Guideline-directed medical therapy, IHD: Ischemic heart disease, NIT: Noninvasive testing, RCT: Randomized controlled trials, CABG: Coronary artery bypass graft, MBF: Myocardial blood flow, CFR: Coronary flow reserve, MVD: Microvascular disease, LMD: Left main disease, SE: Socioeconomic status, MACE: Major adverse cardiovascular events

Identification and Year of Publication	Outcome Measures	Key Findings	Limitations
Li et al. (2022) [3]	This study aims to analyze how the frequency and death rates differ between males and females in different areas and regions of China.	Modifiable risk determinants include high blood pressure, diabetes, physical activity, smoking, alcohol, nutrition, obesity, education, emotional factors, and cholesterol levels. The death rate and risk of having CVD are greater in females than in males. High blood pressure is the risk determinant that mostly affects the Chinese population with CVD.	The population included in this study is not representative of the whole population in China because people from certain regions did not participate. There is a chance that misclassification of measurements reported by the population themselves occurred.
Nussbaum et al. (2022) [5]	This study focuses on the sex differences related to the symptoms' manifestation, treatment, and diagnosis of CAD.	Females manifest non-obstructive CAD more frequently than males. They usually present atypical symptoms, including back pain, palpitations, etc., which lead to delayed diagnosis of the disease and consequently increase the risk of morbidity and mortality.	The inability to give sex-specific instructions and recommendations is due to the low representation of females in clinical trials for CVD, and not enough information about females with CHD is available.
Negrea et al. (2022) [6]	The aim of this study is to analyze the sex disparities in individuals with non-ST elevation acute coronary syndromes (NSTEACS) in terms of risk determinants and management of the condition. Also, it provides an evaluation of the non-traditional manifestation of NSTEACS.	Compared to males, females were more hypertensive and had greater levels of high-density lipoprotein cholesterol; on the contrary, males smoked more and had greater creatinine levels.	The findings of this single-center study with a small sample size cannot be generalized because the sample was from a small geographical area composed mostly of White communities.
El-Menyar et al. (2011) [7]	This study focused on patients with ACS, the variety of symptoms that they manifest, and how they affect the prognosis and outcomes.	Patients who manifest mostly the non-traditional symptoms of CAD get less evidence-based treatment and have a higher mortality rate. Fewer diagnostic tests were performed on these patients, leading to a delayed diagnosis.	The information used for this study was taken from an observational study. The findings cannot be generalized because they were seen in only a particular ethnic group.
van Oosterhout et al. (2020) [8]	The meta-analysis evaluated gender-based discrepancies in the manifestation of symptoms in individuals diagnosed with ACS.	The odds of having nausea, pain between shoulder blades, exhaustion, jaw pain, dyspnea, and throwing up are greater in females than males. However, the odds for chest pain are inferior in the female gender compared to the other symptoms. Regarding right arm, epigastric, and left arm pain and indigestion, there were no discrepancies. It suggests that symptoms of CAD should not be labeled as “typical” or “atypical".	The information gathered by medical record retrieval might generate bias. There is no relation to age. Exclusion of articles that were in a different language. Restricted only to patients with confirmed ACS and not suspected ACS.
Hema et al. (2016) [9]	The RCT aimed to check if risk evaluation, manifestation, testing choices, and outcomes were different in different genders with stabilized symptoms of presumed CAD.	Chest pain is one of the principal manifestations in both genders. It is more probable that men have “aching/dull” pain and “burning/pins and needles”, and females are more likely to have "crushing/pressure/squeezing/tightness" pain. Women had higher comorbidities than men presenting with CAD. Women had more abnormal electrocardiographic changes than men with acute coronary syndrome.	The discoveries can’t be extrapolated to other populations because the study population was limited to outpatients with stable symptoms with presumed CAD and did not include invasive testing.
Coventry et al. (2011) [10]	The study aimed to analyze gender discrepancies in the symptom manifestations of AMI.	Females tend to exhibit inferior odds compared to men with chest pain. Instead, they exhibit more frequent non-traditional symptoms associated with CAD, such as back and neck pain, exhaustion, and palpitations.	Most studies did not include the location and type of infarction and were excluded from the majority of studies. Many research papers had age restrictions.
Khesroh et al. (2017) [11]	This study aims to analyse the influence of sex on the manifestation, treatment, and death of patients diagnosed with ACS.	At the time of diagnosis with ACS, females were older and had more comorbidities than males. They also tend to present with non-traditional symptoms, in contrast to males.	The study only includes citizens from the Gulf area and doesn’t take into account the expats, who make up a large proportion of the population in this geographical area.
Perdoncin et etal. (2017) [12]	The study aims to analyze the sex-specific discrepancies in the management of CAD and how they influence the results for women.	Women usually present with symptoms that are not typical of CAD, like exhaustion, pain in the jaw, and shortness of breath. Women frequently have more complications linked to MI than men; this includes shock, hemorrhage, and stroke. Also, the cardiovascular aftermath for females is worse than that for males.	Absence of acknowledging any limitations. Doesn't specify the inclusion and exclusion criteria.
Sarma et al. (2019) [13]	To analyze the association of sex with major adverse cardiovascular phenomena (CV death, stroke, and MI) as well as all-cause mortality, adjusting for relevant risk factors in individual trials	Women present with CAD at an older age and greater comorbidities (DM, HT, CHF, CKD) have more atypical symptoms and are less treated with GDMT. Women were more likely to have non-obstructive CAD.	Residual confounders that could explain the differences between sexes can’t be excluded. Trials differed in design, time, follow-up, etc. Even though it was multinational, it was predominately White patients.
Du et al. (2017) [14]	Test key performance indicators reflecting in-hospital control of ACS in both male and female patients (including the rate of coronary angiography, PCI, use of evidence-based medications, and major adverse CV events).	Females with NSTEMI had less treatment in contrast with males and received less GDMT. In comparison to males, females have more comorbidities and are older. A greater proportion of women were retired and had lower socioeconomic status and education.	The study did not record the hold-up in hospital presentation or relocation to a specialist. Residual confounding. Although the scale of the study is large, the investigation of the intervention effect isn’t enough to rule out differences in performance and clinical results.
Vasiljevic-Pokrajcic et al. (2016) [15]	Evaluated how CAD differs between sexes in terms of prevalence, treatment received, comorbidities, and outcomes.	Women have a higher range of comorbidities compared to men. Women receive fewer adequate treatments for acute coronary syndrome (4% less than men).	It was performed using only three tertiary medical centers located in Serbia.
Ferrari et al. (2013) [16]	States the main differences between sexes regarding baseline comorbidities and treatment in patients who had stable CAD.	Females were shown to present with a higher burden of comorbidities and risk factors than men. Females had a lower probability of undergoing interventional treatment and receiving coadjutant medical treatment for CAD prevention. It was shown that women have a shorter duration of suffering from CAD than men.	The study did not investigate how sex influenced prognosis in a long-term evaluation. It also did not evaluate other possible confounders that might explain differences in the management of CAD.
Mega et al. (2010) [17]	The study evaluated the clinical, biomarker, angiographic, and continuous ECG characteristics and post-360-day results of females with unstable ischemic heart disease randomized to ranolazine or placebo in MERLIN-TIMI 36.	Troponin values were lower at baseline for women. Women had higher BNP levels at baseline. The ECG of females showed ST depression and higher concentrations of BNP. Women had a median duration of evidence that was longer than men (40 minutes on average). A study showed that women had higher odds of presenting angina than men. Women had higher odds of having more comorbidities when presenting with CAD.	Films of realized angiographic assessments and angiographic evaluations were not checked by an angiographic core laboratory.
Otaki et al. (2015) [18]	The prospective, multinational registry examined the frequency, severity, and composition of coronary artery disease (CAD) in young individuals undertaking coronary CT angiography (CCTA).	Men had a higher prevalence of any kind of CAD in comparison to women.	The study was conducted on individuals who were already suspected of having CAD, which could represent a selection bias.
Smaardijk et al. (2020) [19]	The study evaluated the risks of psychological determinants for IHD incidence in both sexes.	Women present more frequently with non-obstructive IHD, including spasms, takotsubo cardiomyopathy, and microvascular coronary dysfunction. Women showed a 21% and men a 37% elevation in the risk of psychological determinants for MACE.	Miscellaneous studies were encountered. Possible biased publications
Pagidipati et al. (2019) [20]	The RCT measured gender discrepancies in the results of noninvasive testing (NIT) and the medical treatment (aspirin or statin use) that follows in patients with stable symptoms indicative of CAD.	Women received fewer statins, it was more improbable for them to have adverse cardiovascular results in contrast to males, and they were indicated less often for catheterization than men.	Randomization wasn’t categorized by patient sex; nonetheless, within each sex, the two testing arms were alike.
Guo et al. (2018) [21]	Compared outcome differences in both sexes after PCI in a one-year follow-up.	Women had the worst outcome after PCI, and age was an important risk factor associated with it.	The studies included were not RCTs. Women were only 25% of all the patients analyzed, which increases the risk of bias. There was a lot of heterogeneity between the studies chosen. Studies eligible were only in English.
Al-Fiadh et al. (2011) [22]	The RCT aimed to identify if there is an early or medium-term risk in the recent period of PCI between females and males that exhibits ACS by making use of a big multi-center PCI registry based in Australia.	The frequency of coronary disease at angiography was inferior in males and females, and less than half of women who indicated an earlier therapy commencement got PCI.	Information for patients who went through CABG or did not have any procedure was not given. Extended follow-ups were initially part of the plan; nonetheless, this was restricted to 12 months only.
Mehta et al. (2012) [23]	The retrospective observation focused on analyzing discrepancies in risk among females and males and assessing connections between sexes, 365-day mortality, and bleeding rates in patients receiving fibrinolysis for STEMI.	Women experienced more bleeding than men. Invasive procedures were less commonly performed on women. Women had a higher incidence of in-hospital hurdles when experiencing bleeding. Once the occurrence of bleeding was considered in the death model, females had an inferior risk of death.	Possibly other factors or coincidence played a role in the discovery of a connection between bleeding and mortality in females and males. The study's results may not apply to those who undergo PCI intervention due to the lack of information about the timing of bleeding for many patients.
Lichtman et al. (2018) [24]	The clinical trial addressed the missing information regarding the recognition and manifestation of AMI signs in younger patients.	The manifestation of AMI signs does not vary much between genders. The most prominent sign is chest pain in both genders; however, females also manifested other symptoms such as pain in the neck, jaw, arms, and palpitations. In comparison with males, females with STEMI did not show signs of chest pain. NSTEMI was more frequently exhibited in females.	Patients who passed away before hospitalization and did not agree to it were excluded. The study was unable to determine the initial or main symptoms reported by the patients.
Tamis-Holland et al. (2013) [25]	The RCT measures were death, myocardial infarction, cerebrovascular accident, chest pain, and Duke Activity Status Index scores by doing a comparison using the variables, interventions performed, and outcomes between both sexes.	Women had higher odds of presenting with angina. Men tend to have higher rates of left ventricular dysfunction than women. Women had higher odds of presenting with chronic heart failure and having diabetes for a longer time than men, as well as hypertension.	The study did not assess the severity of symptoms, had some missing data, and did not evaluate certain factors such as transportation and the living situation of patients.
Diercks et al. (2010) [26]	The study aimed to show if the “National Women's Cardiovascular Awareness Campaign” had a beneficial effect in reducing the time taken between symptom onset and hospital arrival in women suffering from myocardial infarction.	The average time that women take to present to the hospital after the onset of symptoms is higher compared to men. Women tend to arrive at the hospital after 12 hours of symptom onset. Possible risk factors associated with a longer duration of the interval included older age (being older than 60 years), race, and comorbidities like diabetes and high blood pressure.	Some patients had missing information regarding the time of symptom onset and arrival at the hospital. The study did not perform an evaluation of other factors involved, like transportation, the hospital’s closeness, or other living situations that might affect the patient. Patients who showed up after 24 hours from symptom onset were not included in the study. The study did not show a link between mortality and delay.
Gimenez et al. (2014) [27]	The RCT was designed to show differences in the treatment of suspected myocardial infarction in women by addressing angina’s characteristics and variations between men and women.	Women had higher odds of presenting symptoms of pressure like pain, dyspnea, pain that increased with palpation, pain moving to the neck or the back, sudden onset of angina, or pain that lasted for longer than half an hour. Women had lower chances of having no pain radiation, radiation to the right side of the chest and neck, or pain lasting 2 to 30 minutes.	The study did not evaluate the accompanying symptoms. It presented a bias in favor of typical chest pain.
Serruys et al. (2018) [28]	The multinational trial analyzed death (all-cause), myocardial infarction, or cerebrovascular accidents over 3 years after invasive intervention in the context of the left main disease. Secondary endpoints included events happening between the first 30 days and 3 years after the intervention, as well as outcomes for each sex with left coronary disease.	The female sex had a higher risk profile than the male sex but showed less complexity in coronary anatomy. Women also had greater chances of completing revascularization after percutaneous coronary intervention. However, women who underwent percutaneous coronary intervention had a higher risk of complications, including ischemic and bleeding complications. Sex was not independently associated with the primary endpoints or death after 3 years.	The subgroup used in the study is not a significant sample. The results found should be used to generate a hypothesis regarding the differences.
Xi et al. (2022) [29]	This systematic review and meta-analysis aimed to analyze the existing evidence on how sex affected mortality (short- and long-term) in patients presenting with myocardial infarction with ST-segment elevation.	Mortality (short-term) was higher in women with myocardial infarction than in men. Mortality (long-term) had similar results for both sexes. Both results were shown after adjusting for baseline risk factors.	The study did not consider how sex change affected other outcomes, such as disability, quality of life, and functional status, and relied on previous data taken from observational studies, which may lead to bias.
Berry et al. (2018) [30]	This randomized controlled trial aimed to investigate major ischemic, cardiovascular, and cerebrovascular events such as myocardial infarction or stent thrombosis.	Women who were on dual antiplatelet therapy after one year had a similar risk of ischemia and bleeding complications when compared to men after coronary percutaneous intervention and angioplasty.	The comparisons between treatments in both sexes were posterior to intervention, and this might limit the statistical significance.
Chichareon et al. (2020) [31]	The randomized controlled trial assessed the effectiveness of the treatment by measuring all-cause mortality and new Q-wave myocardial infarction 2 years after PCI. Additionally, the trial evaluated the treatment's safety by monitoring incidents of Bleeding Academic Research Consortium type 3 or 5 bleeding.	The risk of death for any cause was the same for males and females, as was the appearance of new Q-wave MI during a two-year follow-up. Hemorrhagic complications occurred at a higher rate than in men. When analyzing the antiplatelet therapy, the use of ticagrelor monotherapy resulted in a lower risk of bleeding in men over a period of one year.	The study is limited by the lack of stratification by sex, as the female group was smaller than the male one. Also, for higher statistical significance, adjusted analyses should be done.
Lauet al (2017) [32]	This meta-analysis aimed to identify major adverse cardiovascular events, myocardial infarction, stent thrombosis, cardiovascular or all-cause mortality, and bleeding complications after P2Y 12 inhibitor administration.	The use of P2Y12 inhibitors was similar between male and female patients, and in both cases, it reduced the risk of major cardiovascular events, myocardial infarction, and stent thrombosis. The study also found that the use of P2Y12 inhibitors had a similar increased risk of bleeding complications in both sexes.	The study is limited by heterogeneity given by variation among the types of study designs and populations.
Firouzabadi et al. (2013) [33]	The case-control study from Iran investigated the relationship between six gene polymorphisms of renin-angiotensin system compound components and coronary disease.	It was found that there was an independent association between angiotensin-converting enzyme A-240T polymorphism and a higher risk of presenting CAD in Iranian women.	There might be heterogeneity in studies regarding sample inclusion and exclusion criteria, or it might be due to the different racial characteristics of the population taken.
Haas et al. (2019) [34]	This post hoc study examines if there are sex differences in myocardial blood flow (MBF) and coronary flow reserve (CFR) between patients with type 2 diabetes mellitus who do not present clinical indications of obstructive CAD.	In contrast with males, females have poorer blood flow through the myocardium and diastolic function. Rest MBF is related to poorer diastolic function in females; this might be due to the response to aldosterone.	The study is not large, and the initial research did not intend to evaluate the effects of gender. Sex hormones were not considered in this study.
Hokimoto et al. (2014) [35]	The case-control study analyzed the relationship between CYP2C19 polymorphism and the development of coronary artery disease (CAD) when dyslipidemia, diabetes, and chronic kidney disease are not present to reduce the impact of conventional coronary risk determinants.	CYP2C19 PM is a predictor of CAD risk in females alone, but not in males.	The study only investigated the association in a Japanese population and may not be generalizable to other populations. The study did not investigate the functional significance of the CYP2C19 polymorphism or its potential mechanisms of action in the development of CAD.
Gijsberts et al. (2015) [36]	The observational study explored the relationship between set biomarkers and the severity of CAD in stable patients who will undergo coronary angiography, taking into account sex.	The severity of CAD is inferior in females in contrast to males, based on coronary angiography. The relationship between biomarkers and CAD severity doesn’t change between the two sexes.	It was not possible to continue assessing patients for the occurrence of cardiovascular events.
Guimarães et al. (2017) [37]	The study objective is to analyze the relation between gender and clinical results, as well as the relation between sex psychosocial features and cardiovascular risk.	Regarding psychosocial determinants, results, and clinical features, females and males with stable CAD exhibit many discrepancies. Females have more comorbidities and a poorer quality of life. Women have better lasting clinical results; this may be due to the frequency of signs of depression; the more prevalent these symptoms were, the more chances the cardiovascular risk of women was similar to that of men.	The number of females that participated in this study is shorter than that of males. Rigorous inclusion criteria were used, so the pool of individuals might not be a full representation of subjects diagnosed with stable CAD. A strict psychological assessment wasn’t conducted.
Norris et al. (2008) [38]	To compare HRQOL results post-cardiac catheterization to verify if sex discrepancies persist following adjustment of set risk determinants, baseline HRQOL, symptoms of depression, and social assistance among CAD patients of both sexes. Also, this analysis evaluated whether these determinants explain sex differences in HRQOL.	Females with coronary artery disease presented a poorer quality of life associated with health 365 days after coronary angiography, in contrast with males. The Seattle Angina Questionnaire scores are considerably more elevated in males than females.	The study pool of individuals was restricted to patients catheterized for CAD and made a response to the baseline and the 365-day follow-up. Additionally, the study did not retain the medication that was given and utilized over the 365 days.
Dreyer et al. (2015) [39]	The goal of this study is to analyze sex discrepancies in health status through time with a baseline of up to one-year post-AMI. The study analyzed mental and physical functioning, quality of life, and angina. It also analyzed recovery time and the impact of sex on each health status.	They had unfavourable physical restrictions, quality of life, and mental performance. The recovery time was alike between both sexes; however, females in the same age range as males who had an episode of AMI had a lower score in the entire health status.	Patients did not provide a response for the follow-up interview, and a portion was lost. The study could not reach patients who were sick and couldn’t participate. The study was observational, and the discrepancies among both sexes in health status might be due to measurement errors.
Orth-Gomér et al. (2018) [40]	The study measured the effect of psychological, clinical, and social determinants concerning the social degree of CAD patients with depression in a Germany-based trial.	Social support varied by sex and education level. Women with low education received the least support, while men with academic backgrounds received the most. Depression was highest for both genders in the lowest socioeconomic group, but significant only for women.	The description and categorization of socioeconomic status (SES) should be considered while drawing conclusions. The grouping of SES is a delicate matter, and several proportions of SES are outlined, with education being the more frequently utilized criterion for SES.
Daoulah et al. (2017) [41]	The observational, multicenter study focused on establishing the relationship between divorce and the severity of CAD, MVD, and LMD in men and women.	Critical CAD, MVD, and LMD are related to various divorces in females but there is no relation with males.	The number of divorcees is not large enough, and the time between the separation and cardiac catheterization wasn’t recorded. Generalizations of findings to divorcees in an unaffected population cannot be made.

Influence of age on CAD

Age-Related Prevalence and Risk of CAD and Differences in CAD Presentation Across Different Age Groups

As individuals grow older, the severity and incidence of CAD increase, with those over 75 years of age being more likely to have multi-vessel CAD [42]. Patients aged above 65 years old have greater major adverse cardiovascular events (MACE) risks for non-obstructive CAD with one or two vessels affected, in contrast to normal patients below 65 years of age who present with three vessels and left main (LM) disease and have a greater risk for MI and late revascularization. The degree and severity of MACE were more elevated in patients over 80 years old.

Atherosclerosis begins at a younger age and is influenced by various factors, primarily abnormal lipid metabolism [43]. Unhealthy eating habits, smoking, alcohol consumption, obesity, sedentary lifestyles, and a family history of CVD can lead to premature coronary disease (PCAD) in young, below-35-year-old individuals who live in rural areas. This might be because in these areas there are more underprivileged patients, and they lack awareness or higher education, which could contribute to the rise of PCAD.

The quantity of lipoprotein (a) (Lp(a)) obtained in the adult period is from when the individual was five years old [44]. Raised volumes of Lp(a) are a critical determinant of CVD associated with atherosclerotic plaque, particularly in patients and other individuals with familial hypercholesterolemia.

Coronary artery calcium (CAC) is a tool used to estimate atherosclerotic burden in both younger and elderly patients [45]. A study verified the chances of the CAC score being greater than 0 for adults between 30 and 45 years of age, and this was different across individuals with different age ranges, sexes, and races: 34-year-old White males were in the 90th percentile in contrast to 37-year-old Black males with CAC >0, and the overall chances of having CAC >0 increased with age in both races (White and Black) and both sexes. Studies also linked a greater incidence of CAC with an increased number of atherosclerotic cardiovascular disease (ASCVD) risk factors [45].

Age-Specific Outcomes and Prognosis of CAD

In terms of therapy, decreasing the volumes of Lp(a) possibly improves the outcomes of younger patients diagnosed with AMI since it has been seen that reducing the amount of Lp(a) reduces MACE [44]. In a community of people in their 70s, a multifaceted prevention scheme was linked to a diminished risk of ischemic heart disease, more specifically, a 13% lower risk of CVD. Two to five years after the scheme was initiated, the risk of CVD decreased by 20%. Among participants who were at higher risk, the preventative program was linked to improved hypertension and hypercholesterolemia treatment [46]. The number of unnoticed AMI diagnoses and the billing of care for individuals ≥ 65 showing up at the emergency unit varied in different hospitals. These individuals were released earlier, after their visit and admission to the hospital [47].

Discussion of Potential Biological and Age-Related Factors

The shortening of telomeres is an indication of the aging process, and it also contributes to atherosclerosis, thus affecting CVD [48]. As individuals get older, the telomeres get shorter and shorter. In an RCT, it was found that in patients diagnosed with MI at an average age of 75 years, leukocyte telomere length (LTL) is connected to linoleic acid concentration. However, this connection is not observed with other long-chain polyunsaturated fatty acids (LCPUFA) in the serum. The trial also revealed that patients with a more balanced nutrition diet seem to have extended telomere [48]. The summary of the findings from the referenced studies on the influence of age as a non-modifiable risk factor on CAD is in Table 2.

**Table 2:** The impact of age on CAD CAD: Coronary artery disease, CVD: Cardiovascular disease, CCTA: Coronary computed tomography angiography, MACE: Major adverse cardiovascular events, LM: Left main, MI: Myocardial infarction, PCAD: Premature coronary artery disease, HDL: High-density lipoprotein, Lp(a): Lipoprotein (a), CAC: Coronary artery calcium, AMI: Acute myocardial infarction, LTL: Leukocyte telomere length, LCPUFAs: Long-chain polyunsaturated fatty acids

Identification and Year of Publication	Outcome Measures	Key Findings	Limitations
Nakazato et al. (2014) [42]	The observational study examined the relation between the degree and severity of coronary artery disease (CAD) through CCTA and the risk and frequency of major adverse cardiac occurrences with respect to age.	Patients aged above 65 have greater MACE risks for on-obstructive CAD with one or two vessels affected, in contrast to normal patients below 65 years of age. Patients below 65 years of age with three vessels and LM disease have a greater risk for MI and late revascularization. The degree and severity of MACE were more elevated in patients over 80 years old.	Selection, referral, and misclassification biases are possible.
Patil et al. (2020) [43]	The observational study evaluated the risk determinants and clinical and angiographic picture of young individuals from India living in rural areas showing premature coronary artery disease (PCAD).	Traditional risk factors, including decreased HDL volume, smoking, and abdominal obesity, have a significant impact on the development of early coronary artery disease in young people in rural areas.	Dietary patterns of rural youth were not known and may have thus impacted the development of CAD.
Stătescu et al. (2023) [44]	This systematic review evaluated the classic risk determinants of myocardial infarction in the “young”, emphasizing the clinical ramifications of lipoprotein (a).	The occurrence of acute myocardial infarction is rising in younger individuals. Raised volumes of Lp(a) are a critical determinant of the risk of cardiovascular disease associated with atherosclerotic plaque, particularly in patients and other individuals with familial hypercholesterolemia. Treatments that decrease the volumes of Lp(a) may improve the outcomes of younger patients diagnosed with AMI.	The study fails to determine the exact impact of Lipoprotein A (Lp(a)) lowering agents on the medical management of CAD and relies on the ongoing Phase 3 trial of a Lp(a) lowering agent in a randomized, double-blinded placebo-controlled trial.
Javaid et al. (2022) [45]	The study determined the probability of coronary artery calcium (CAC) >0 and developed age-sex-race percentiles for U.S. adults aged 30–45 years.	The incidence of CAC in white men was >0 of 26%, Black males of 16%, White females of 10%, and Black females of 7%. Women were put at the >90th percentile with a CAC >0. 34-year-old White men were in the 90th percentile in contrast to 37-year-old Black men, according to CAC >0.	The study did not have data on East Asians, Hispanics, South Asians, or other races and ethnicities. Any long-term clinical outcomes were also not studied.
Nordström et al. (2020) [46]	The cohort study assessed the risk of CVD in a main prevention scheme for a community of people in their 70s.	In a community of people in their 70s, a main multifaceted prevention scheme was linked to a lower risk of stroke and ischemic heart disease. Among participants who were at higher risk, the preventative program was linked to improved hypertension and hypercholesterolemia treatment.	Randomization was not done due to ethical reasons. The study could only guess if any effects on CVD are attributable to alterations in blood pressure and cholesterol volumes as a result of improved medication and/or behavioral changes as a result of the motivational interview, and no proof of causal effects could be established.
Wilson et al. (2014) [47]	The observational study measured the differences in unnoticed diagnosis and billing of care for aged individuals with acute myocardial infarction (AMI) who show up in the emergency unit. Also, this study aimed to recognize the emergency unit and hospital features connected to the differences.	The number of unnoticed acute myocardial infarction (AMI) diagnoses and the billing of care for individuals equal to or above the age of 65 showing up at the emergency unit varied in different hospitals. These individuals were released earlier after their visit and admission to the hospital.	The study relied on administrative data only and utilized a short index of coding for medical release diagnosis of conditions indicative of cardiac ischemic diseases, thus underestimating the true incidences of unnoticed AMI cases. The billing of care was studied only from the payer's point of view and not from the hospital’s or the patient's point of view.
Kalstad et al. (2019) [48]	The randomized controlled trial explored the interconnections among serum polyunsaturated fatty acids, leukocyte telomere length, serum, cardiovascular risk determinants, diet, and characteristics of myocardial infarction (MI) in senior patients.	In this analysis, there was a small connection between linoleic acid but no considerable relation between LTL (leukocyte telomere length) and marine LCPUFAs (long-chain polyunsaturated fatty acids) has been identified. Extended telomeres seem to be associated with balanced nutrition.	The study doesn't have a control group of individuals within a similar age range without CVD risks. Medication taken previously for the index infarction may have affected the outcomes.

Influence of race and ethnicity on CAD 

Racial Disparities in CAD Prevalence and Risk

Race is determined by dividing people into groups according to their physical features and providing groups with social significance, whereas ethnicity is determined by the social and cultural groups a person belongs to. Significant differences between CAD and stroke epidemiology and outcomes exist based on racial and ethnic factors and their discrepancies. Despite significant advancements in these domains, subgroups of racial and ethnic minorities have been marginalized in clinical trials and population data, which makes it difficult to fully comprehend these inconsistencies.

The prevalence of CAD in Black men is lower (5.7%) compared to non-Hispanic White (NHW) men (7.9%), whereas in Black women it is higher (5.2%) than NHW women (3.2%) [49]. In a different study, the prevalence of CAD was lower in Hispanics (5%) than in NHW and Black patients, on the grounds of inaccurate death records, underrepresentation, and misclassification of deaths [50]. In Puerto Ricans, CAD was the leading cause of death, equivalent to NHW individuals. The rates of CAD mortality were the lowest in Mexicans when compared to NHW, Cubans, and Puerto Ricans [51]. The prevalence of CAD is lower in Asian males (4.8%) and females (3.2%) compared to all groups mentioned earlier [50]. Asian Indians and Filipino males have the highest proportionate mortality from CAD, with significant rates of early CAD in both populations. After reviewing over 94,000 electronic health records, it was found that Asian Indian men and Filipino men and women (odds ratio of 1.77, 1.47, and 1.66, respectively) have greater rates of CAD compared to the NHW group. Moreover, Chinese people had a lower prevalence of CAD in general [52].

Differences in CAD Presentation Among Different Races

Asian ethnic groups present with a history of previous transient ischemic stroke, peripheral arterial disease (PAD), coronary artery bypass grafting (CABG), cerebrovascular accident, and acute coronary syndrome (ACS) [53]. In comparison to the White population, the Chinese had a more pronounced association between male gender and more severe CAD (OR 7.0 (4.0-12.6), p-value for interaction = 0.001). When comparing the triple vessel disease occurrence, the prevalence was highest in Malays (31.6%), then the Chinese (23.8%), followed by the Indians (23.2%). The severity of CAD was higher and independently associated with Chinese and Malay ethnicities compared to the White population. Consequently, other above-mentioned ethnicities exhibit greater CAD severity, so the screening process for these races should be expedited. Around 11% of White patients had a STEMI when they arrived at the angiography lab, followed by Malay (8%), Chinese (7.6%), and Indian (7.5%) patients. Chinese people, Indian people, and Malay people had an increased rate for the unified category of non-ST-elevation myocardial infarction (NSTEMI) or unstable angina (UA) than White people. So, White patients demonstrated higher levels of STEMI, whereas the other groups of patients presented with NSTEMI [53].

All three genetic models demonstrated a substantial correlation with CAD in four out of the five ancestral groups (except Africans). While comparing the associations, the Middle Eastern group displayed the greatest levels (for all comparisons, Z value range = 2.92-3.94, p = 0.004), followed by the European group (for all comparisons, Z value range = 2.10-3.37, p = 0.04). So, Middle Eastern groups had the highest prevalence of CAD among the genetic models. Asians consistently demonstrated the highest level of connection across all three models, followed by Europeans [54].

Race-Specific Outcomes and Prognosis of CAD

Mortality and morbidity rates of ischemic heart disease in those who identify as racial or ethnic minorities are excessively high. This can be attributed to the potential explanation that patients from minority groups are unlikely to get coronary revascularization or guideline-directed medical therapy done, according to studies on treatment inequalities. Additionally, Black and Hispanic patients had reduced statin therapy adherence (50.3% and 58.6%) when compared to White and Asian patients (67.4% and 72.3%). Black patients exhibited greater all-cause mortality over a median follow-up of 7.5 years (unadjusted hazard ratio = 1.88, 95% confidence interval = 1.09-3.24) compared to White patients [55]. No difference was noted in revascularization rates and initial medical treatment between racial and ethnic groups, but long-term medication adherence did, with Black and Hispanic patients being more likely to take their medications as prescribed.

In comparison to White men, the risk for fatal CHD was higher in Black men, whereas the risk for nonfatal or total CHD was similar or lower after keeping age as a control. On the other hand, there was a greater risk of fatal, nonfatal, and total CHD in Black women than White women, particularly among those over 65 [49]. Cubans and Puerto Ricans exhibited CVD death rates that were equivalent to NHWs after age adjustment [51]. Survival rates following CAD events were similar in all ethnic groups except Malay, which showed a comparatively higher mortality rate primarily due to more conservative treatment and less use of preventive medicines [53].

Black patients had more comorbidities like diabetes, hypertension, and modifiable risk factors like smoking and obesity than White patients. In this matter, it was found that percutaneous coronary intervention (PCI) for Black patients exhibited a comparable likelihood of in-hospital survival post-procedure to White patients. However, when followed up for 30 days and one year, the mortality was higher in the Black patient group [56].

Discussion of Potential Genetic Factors

The CYBA gene is associated with making a protein called cytochrome b-245 alpha chain (or p-22phox). This protein forms an enzyme called NADPH oxidase, which is an important source of reactive oxygen species (ROS) involved in the pathogenesis of CAD. The T allele for this gene, when compared to the C allele, leads to decreased levels of low-density lipoprotein (LDL) cholesterol and hence further decreases CAD severity. When compared to Asians (8.7% cases, 9.5% controls), the White population has a much greater frequency of the T allele (34.3% cases, 33.5% controls) [57]. The polymorphism of CYBA C242T is linked to CAD.

Nitric oxide synthase (NOS) is an important factor in vascular regulation. Genetic polymorphisms of the NOS gene can lead to irregularities in the vasculature. The global ancestry-spanning relationship between CAD and the three prevalent NOS3 gene polymorphisms, Glu298Asp, T786-C, and 4a/b VNTR, has been studied. The Middle Eastern group exhibited the strongest correlation with both the examined NOS3 SNPs, Glu298Asp and 4b/a. However, the greatest risk for CAD among individuals of Asian descent appears to be associated with T786-C and its minor allele [54].

**Figure 2:**
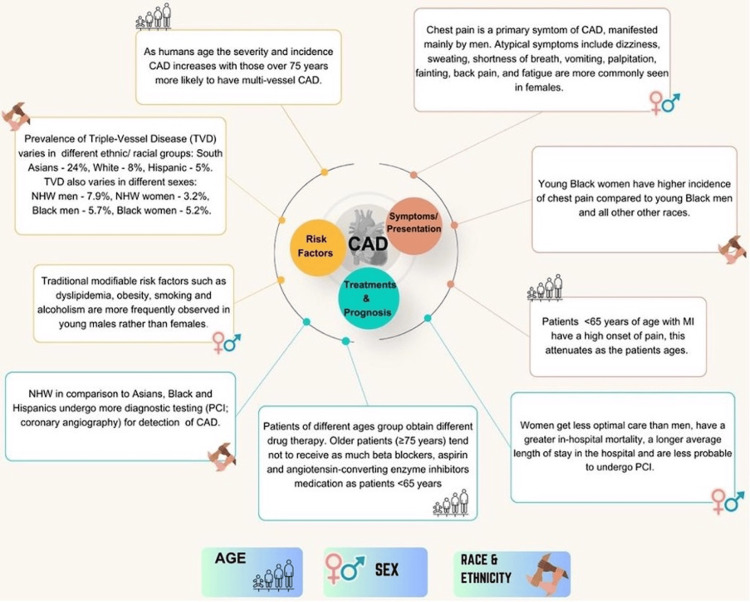
Overview of the intersectionality of non-modifiable risk factors, i.e, sex, age, race, and ethnicity on the risk, presentation, and outcomes of CAD CAD: Coronary artery disease, PCI: Percutaneous coronary intervention, NHW: Non-Hispanic White, TVD: Triple-vessel disease Image created by authors and based on the data from the studies included in this review.

Apolipoprotein E (ApoE) plays a significant role in metabolizing cholesterol and triglycerides. Lower levels of cholesterol and high triglyceride levels are associated with the E2 allele, and increased levels of total and low-density lipoprotein cholesterol levels are observed in carriers of the E4 allele. According to the data, a lower risk of CHD was associated with the ApoE2 allele, whereas the ApoE4 mutation was linked to a higher risk of CHD in White people [58]. During the process involved in the formation of atherosclerotic plaques, CDKN2A, a tumor suppressor gene, has a vital role in regulating the cell cycle checkpoints. Numerous studies have been conducted on the rs1333049 polymorphism of CDKN2A to see whether it may be linked to CHD. In both Europeans and Asians, the rs1333049 polymorphism has been found to dramatically increase the risk of CHD [59]. An overview and summary of the findings from referenced studies on the influence of ethnicity as a non-modifiable risk factor on CAD are in Figure 2 and Table 3, respectively.

**Table 3:** The association of ethnicities with CAD CAD: Coronary artery disease, CHD: Coronary heart disease, ARIC: Atherosclerosis Risk Communities study, CHS: Cardiovascular Health Study, REGARDS: Reasons for Geographic and Racial Differences in Stroke study, CVD: Cardiovascular diseases, NHW: Non-Hispanic White, PVD: Peripheral vascular disease, DM: Diabetes mellitus, AMI: Acute myocardial infarction

Identification and Year of Publication	Outcome Measures	Key Findings	Limitations
Mital et al. (2021) [49]	The article reviews the ethnic and racial discrepancies persisting in the care of patients with CAD and stroke.	CAD predominantly affected the Black population. Racial discrimination and CAD may be linked for decades; therefore, racial prejudice in childhood or adolescence may increase the risk of CAD in adults. Unjust treatment based on race in childhood is linked to an increased cumulative chronic biological risk.	This is a review study and, hence, doesn't establish a cause-and-effect relationship. The psychological, social, and environmental factors pertaining to different racial and ethnic groups need to be further evaluated. Lack of sufficient data and underrepresentation of minority groups further diminish the true results of the study.
Colantonio et al. (2017) [50]	In the Atherosclerosis Risk Communities study (ARIC), the Cardiovascular Health Study (CHS), and the Reasons for Geographic and Racial Differences in Stroke study (REGARDS), the fatal and nonfatal CHD incidence and CHD case-fatality were compared among Blacks and Whites by sex.	In comparison to White men, the risk for fatal CHD was higher in Black men, whereas the risk for nonfatal or total CHD was similar or lower after keeping age as a control. On the other hand, there was a greater risk of fatal, nonfatal, and total CHD in Black women than White women, particularly among those over 65. In comparison to White men and women, the risk of fatal CHD was similar in Black men and women, whereas the risk of nonfatal and fatal CHD was reduced after keeping social determinants of health and cardiovascular risk factors under control. It was also found that social determinants of cardiovascular risk factors and health could not explain the higher case-fatality rates in the Black population when compared to the White population, including both men and women.	The three cohorts used different data collection methods. Not many non-White and non-Black participants in ARIC were included in the analysis, as non-Whites and non-Blacks are indistinguishable from Whites using the publicly accessible dataset. Results from ARIC and CHS may not be generalizable to the entire US population due to their restricted geographic coverage. In REGARDS, nonfatal CHD occurrences are adjudicated based on participants who self-reported CHD-related hospitalization, which may lead to an underestimation of incidence rates.
Rodriguez et al. (2017) [51]	The multicenter study included the three largest US Hispanic subgroups, i.e., Mexicans, Puerto Ricans, and Cubans, and aimed to articulate a decade of national CVD (cardiovascular diseases) mortality data.	Younger patients were seen in Puerto Ricans and Mexicans than in Cubans and NHWs when it came to CVD deaths. Cubans and Puerto Ricans exhibited CVD death rates that were equivalent to NHWs after age adjustment but overall had higher rates of ischemic and hypertensive heart disease. Higher mortality rates from cerebrovascular illness were seen in Mexicans than those of other Hispanic groupings but remained lower than those of NHWs.	Further disaggregation of a heterogeneous population of Dominicans, Spaniards, Latin Americans, and South and Central Americans that were included in the comparison groups of Hispanics could not be accomplished owing to inconsistencies in data collection. Certain discrepancies are possible in mortality data. Factors underlying the racial and ethnic differences, like area of residence, acculturation metrics, health behaviors, etc., were not considered.
Holland et al. (2011) [52]	The study includes Asian-American subgroups (Asian Indian, Filipino, Chinese, Japanese, Vietnamese, and Korean) and NHW subjects to compare the prevalence of coronary heart disease (CHD), peripheral vascular disease (PVD), and stroke in a mixed-payer, outpatient health care organization in California.	The risk for CHD was higher in Asian Indians and Filipinos, whereas an elevated risk for total stroke was seen in Filipino women. The risk of ischemic and hemorrhagic stroke in Filipino women was significantly higher than that of NHWs. Chinese men and women had a much lower risk of CHD when compared to NHWs. The ORs for CHD and stroke are close to one and not statistically significant because certain subgroups have higher rates than NHWs and others have lower rates when comparing the Asian group to NHWs. In contrast, the probability of PVD decreased across the board for all Asians. The prevalence of traditional risk factors for CAD, like type 2 DM and hypertension, is higher in Filipinos and Asian Americans.	The study's limitations include a specific geographic area and a very small sample size in the Asian subgroups (i.e., Korean and Vietnamese populations). The prevalence definitions of CVD were built on outpatient visits only, which might have led to an underestimation of CVD prevalence.
Gijsberts et al. (2015) [53]	The study explores the interactions between risk factors and ethnicity on the CAD severity of four ethnic groups: White, Chinese, Indians, and Malay, while using multivariable ordinal regression. Also, multivariable Cox regression analysis was used to compare all-cause mortality among the ethnic groups.	Indians (South Asians) tend to develop CAD earlier in life. CAD severity was higher and independently associated with Chinese and Malay ethnicities compared to the White population. The risk factor burden was found to be higher in Chinese, especially due to diabetes. Survival rates following CAD events were similar in all ethnic groups except Malay, which showed a comparatively higher mortality rate primarily due to more conservative treatment and less use of preventive medicines.	There was no correction of confounding factors like diet, lifestyle, and socioeconomic factors. The health care delivery system in Singapore and the Netherlands, where the study was conducted, is different and hence may impact the findings.
Rai et al. (2014) [54]	The comprehensive review and meta-analysis looked at the association between CAD and three polymorphic variants of the NOS3 gene, specifically Glu298Asp, T786-C, and 27bp VNTR b/a. It also examined their connection independently among published studies with a predominance of Middle Eastern, Asian, European, African, and Asian-Indian ancestry in a subgroup analysis.	The research supports the global link of CAD with the three frequent NOS3 gene polymorphisms: Glu298Asp, T786-C, and 4a/b VNTR. The results between various ancestral subgroups were quite enlightening. The Middle Eastern grouping exhibited the strongest correlation with both the examined NOS3 SNPs, Glu298Asp and 4b/a. However, among individuals of Asian descent, the highest risk for CAD is carried by T786-C and its minor allele.	The South Asian region was underrepresented. The association study also doesn't establish causal relationships; it only measures statistical associations. Also, the presence of selection bias, the possibility of errors in genotyping, and the risk of an inadequate sample size cannot be ruled out.
Hammershaimb et al. (2023) [55]	This retrospective study evaluated patients with acute myocardial infarction (AMI) for differences in risk factors, treatment, and outcomes based on race or ethnicity. Patients aged 18 to 50 who were hospitalized for AMI between 2006 and 2016 were included in the study. The association of race or ethnicity with all-cause mortality was evaluated using Cox regression models.	Smoking, diabetes, and hypertension are major cardiovascular risk factors for Black patients, while obesity and diabetes are for Hispanic patients, and smoking is a huge risk factor for White patients. No difference was noted in revascularization rates or initial medical treatment between racial and ethnic groups, but long-term medication adherence did, with Black and Hispanic patients being more likely to take their medications as prescribed.	Only patients who went through coronary angiography were included in the study. Patients who were medically managed for AMI were not part of the study. Ancestral backgrounds were not taken into consideration, although patients were divided into broad racial and ethnic groups. There could be possible errors in measuring medication adherence as information was collected from the pharmacies.
Mochari-Greenberger and Mosca (2015) [56]	The review’s aim is to investigate factors associated with differential outcomes by race and ethnicity among CHD patients by highlighting and evaluating recently published research.	High rates of mortality and hospital readmissions have been seen in minority groups of different races and ethnicities when compared to White individuals. The difference was explained by socioeconomic factors and the presence of other medical conditions.	Additional factors that may contribute to racial and ethnic differences in CHD outcomes have not yet been identified or measured well enough, as disparities existed even after controlling for clinical and socioeconomic conditions. Also, most of the information used in the study was related to Black and White populations only.
Wu et al. (2013) [57]	The comprehensive meta-analysis evaluates the impact of the CYBA C242T polymorphism on CAD and potential biases.	The C242T polymorphism has a heterogeneous influence on CAD among various ethnicities, being moderate in the White population but lacking influence in Asians. Study design and BMI were the two confounding factors responsible for between-study heterogeneity.	The meta-analysis focused on a single variant in the CYBA gene and did not take into consideration the potential synergistic effects of additional candidate genes or polymorphisms. The interplay of polygenic and environmental variables is most likely at the root of genetic susceptibility to CAD, and optimal models addressing these elements are necessary.
Xu et al. (2016) [58]	The meta-analysis aimed to evaluate the effects of apolipoprotein E gene variants on coronary artery diseases across various ethnic groups.	In the stratified analysis, the risk of CAD was high in the Mongolian group and mild in the White group for the allele genetic variation, demonstrating that the ApoEε4 allele contributed to the increased risk of CHD while having differences in genetic background. In addition, the ε2 allele variant demonstrated a lower risk of CHD in White patients but not among Mongolians.	The ApoE gene is a suitable and common gene to study gene-environment interactions. However, a lack of sufficient data limited further evaluation of potential gene-gene and gene-environment interactions.
Lian et al. (2014) [59]	The meta-analysis aimed to establish the link between CDKN2A rs1333049 polymorphism and coronary heart disease (CHD) and its effect on various ethnic groups.	CDKN2A rs1333049 polymorphism contributed to an increased risk of CAD by 38%. CHD risk was increased in both Europeans and Asians by 30% and 27%, respectively, after performing a subgroup analysis by ethnicity showing the association of rs1333049.	Only one variant of the CDKN2A gene was included in the study. Not much information was available for the African population. The results may have been affected due to the small statistical power (p = 0.377) of the case-control study in the subgroup analysis on sex and age.

The combined influence of sex, age, race, and ethnicity on CAD outcomes and prognosis**

Discussion of Potential Intersectional Factors and Their Implication

**Figure 3:**
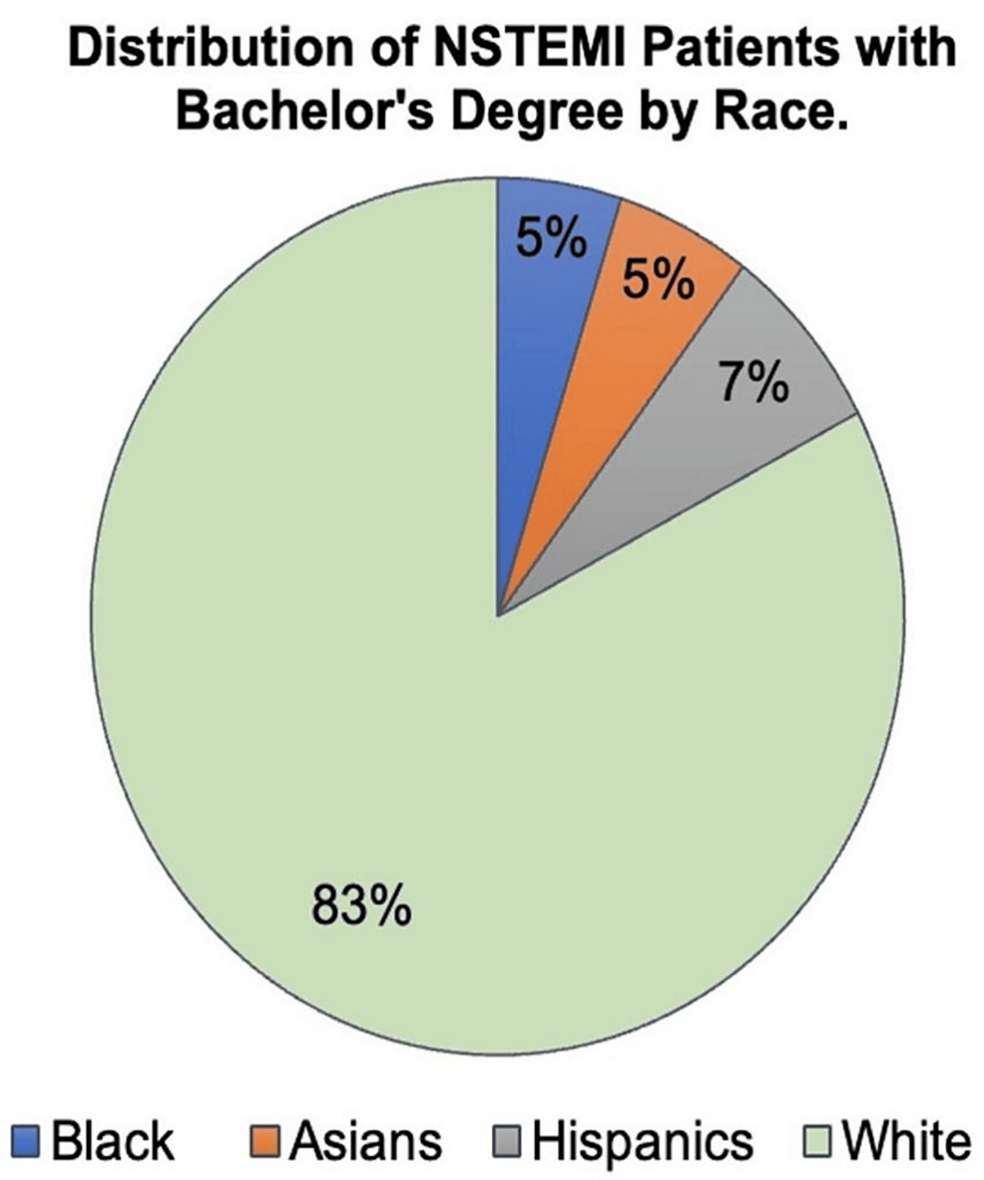
Distribution of NSTEMI patients with a bachelor’s degree by race NSTEMI: Non-ST-elevation myocardial infarction Image created by authors and based on the data from the studies included in this review.

**Figure 4:**
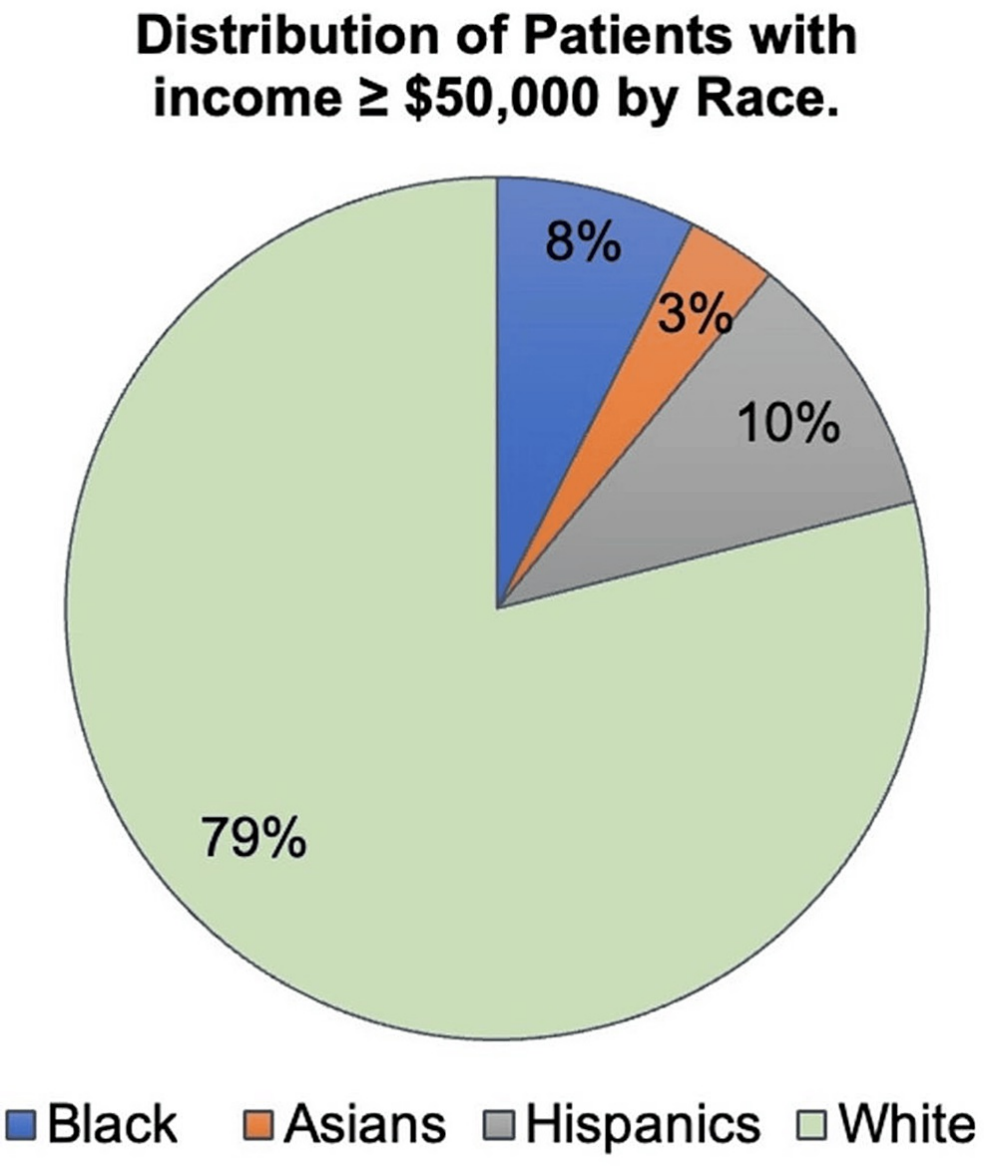
Distribution of patients with income ≥ $50,000 by race Image created by authors and based on the data from the studies included in this review.

**Figure 5:**
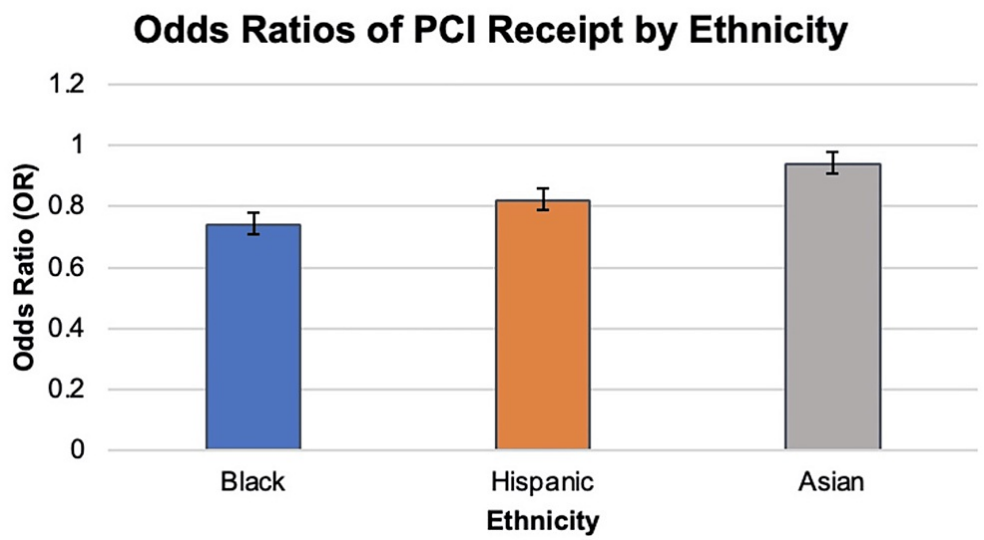
Odds ratios of PCI receipt by ethnicity This figure was created based on the data from other studies adopted by Tertulien et al. [63]. PCI: Percutaneous coronary intervention

As CAD is a multifactorial disease, many factors like age, sex, race, socioeconomic conditions, and education influence it in different ways. Each one of these factors affects its presentation, progression, and severity in a unique manner. The term intersectionality means that an individual experience is due to the compounding effect of various factors [60]. The disparities in severity, prevalence, and outcomes of CAD cannot be fully understood by looking at the above factors individually, thus emphasizing the importance of intersectionality. For example, the experience of a young Black woman with CAD is not only influenced by her age, sex, and race but also by the interaction of all these factors with her social and economic conditions. Black women remain in ICUs for extended periods of time following CABG procedures and have a higher hospital mortality rate when compared to Black men [61,62]. Another study emphasizes that 4.9% of individuals of the Black race have a bachelor’s degree compared to 7% of Hispanics, 5.4% of Asians, and 82.6% of White people (Figure 3) [63]. An annual household income of ≥$50,000 was observed in 7.5% of Black people, 3.4% of Asians, 10% of Hispanics, and 79% of White people (Figure 4). These differences in education and household income affect the diagnosis, outcome, and management of CAD across these groups, as seen in Figure 5.

Age is paramount when considering risk, presentation, and outcomes for patients with CAD. A comparative study showed that as the age of both male and female patients increased, the mortality rate increased as well, regardless of the income of the population [64]. In this study, five illustrative countries, namely the United States, United Kingdom, Kazakhstan, Ukraine, and Brazil, each representing a specific income group, i.e., high, upper middle, and lower middle, were selected, with Ukraine being the country with the lowest income and the greatest mortality rates across both genders when compared to the other four countries. The study also demonstrated a higher male mortality rate compared to females.

To further analyze the age factor, a study demonstrated that patients under 65 years old had a higher onset of chest pain, while in patients above 65 years of age, chest pain was attenuated [65]. There were sex disparities within races and among different ethnic subgroups. Black women presented at a younger age with chest pain when compared to all other races and compared to men; in contrast, Hispanic males had an increased incidence of chest pain than Hispanic females.

Chest pain is a primary manifestation of CAD, identified and assessed in patients; this symptom is not always present, especially among younger women [65-67]. Instead, other atypical symptoms are manifested. A study demonstrated that women under 45 years of age who don’t manifest this hallmark have a higher hospital mortality rate when compared to men under 45 years of age [65]. On the contrary, this sex inequality declines or changes completely with increasing age. Therefore, it is crucial to note that the presentation of CAD can differ from patient to patient and that factors such as age, sex, race, and ethnicity need to be taken into account, so healthcare professionals should be educated about the clinical presentation of typical and atypical symptoms [67]. Early recognition of these atypical symptoms could lead to a faster diagnosis, better management, and a more effective treatment plan, thus possibly reducing the increased mortality risk.

Furthermore, Hispanic patients with AMI have a higher death rate compared to NHW patients, and a study showed that this is associated with insurance coverage, and Hispanic patients have more non-private insurance when compared to NHW patients [68]. Non-private insurance is associated with low income, and these findings suggest once again that economic factors influence CAD, and those who are underprivileged have a higher incidence of CAD.

In terms of the pattern of obstructive CAD, ACS is the most frequently displayed clinical manifestation for both Indian females and males [69]. The STEMI occurs more often, followed by unstable angina, NSTEMI, and the last and least frequently occurring, chronic stable angina. Also, 5.5% of young Indian men had TVD, but this was not seen in young Indian females; instead, they presented more often with single-vessel disease (SVD). Another study also showed that the rate of TVD is 16% higher in South Asians compared to the White population and that the severity of CAD is lower on the ladder [70].

An observational study showed that after undergoing PCI, Black females, in contrast to White females, presented with CAD at a younger age, and target vessel revascularization and stent thrombosis were much greater in Black patients at the one-month and one-year landmarks post-procedure [71]. So far, it has been found that the CAD mortality rate increases with age; nevertheless, CAD is also common in individuals younger than 45 years [69]. Thus, when evaluating the symptoms of younger patients, healthcare professionals should not rule out CAD.

There were also significant differences among sexes and groups of different ethnicities and ages in relation to outcomes, diagnosis, prognosis, and treatment; the result of an observational retrospective analysis emphasizes the difference in outcomes and treatments among Asians in the United States; for instance, NHWs in comparison to Asians overall undergo more diagnostic testing for CAD [72]. Excluding Filipinos, all Asian subgroups received coronary angiography less often than NHWs. One year post-angiography, the percentage of diagnosis of MI was higher (17.4% in Asian Indians and 15.6% in the Chinese, compared to a lower 11.2% in NHWs). Also, Chinese, Filipinos, and Japanese were prescribed more clopidogrel than NHWs after stenting, with the Japanese receiving 91.4% of the prescribed medication. It is also worth noticing that when diagnosed with CAD, all Asian groups except the Japanese were younger in reference to NHWs. By undertaking more diagnostic testing, South Asians can have their symptoms evaluated earlier and therefore receive a faster diagnosis, so a treatment plan can be put in place right away to prevent the exacerbation of the symptoms with time and possibly reduce the increasing mortality rate with age. The standardization of testing could possibly diminish the disparities among different ethnic groups.

Another study emphasizes the racial and ethnic discrepancies during NSTEMI treatment and how social factors such as income impact the likelihood of having those treatments [63]. Patients with inferior income and educational levels have a lower chance of undergoing coronary revascularization. In this study, it was observed that a greater percentage of individuals of the White race had a superior educational background and income compared to the other races and ethnicities (Black race, Hispanic ethnicity). So, when compared to White people, the odds ratio for Black people to have coronary angiography and PCI is lower than 1, more specifically 0.86 for PCI and 0.93 for coronary angiography. Hispanic people have an even lower odd ratio of 0.85 and 0.88 for PCI and coronary angiography, respectively, which means that it is more improbable for Black people and Hispanics to undergo these procedures. Also, a cohort study showed that there is less probability of females going through PCI compared to males, and females had 1.5% elevated in-hospital mortality in contrast to males [61].

Treatments also differ between different ethnic groups with respect to insurance coverage [68]. A study showed that 68.8% of NHWs have private insurance compared to 43% of Hispanics, which results in NHWs having more access to different treatments for CAD. Furthermore, patients with private insurance had a decreased mortality rate in contrast with those with non-private insurance.

Regarding post-CAD care, the quality of care among females and males is not the same, where women get less optimal care than men and have higher mortality rates [73]. Nonetheless, the quality of care does not differ among different ethnicities; however, Black women have higher death rates than White females. In addition, Black female patients who receive CABG remain longer (an average of 27 hours) in the ICU and in the hospital compared to other races after their surgery [62].

Implications for clinical practice and public health

The Importance of Considering Sex, Age, Race, and Ethnicity in CAD Diagnosis and Treatment

As mentioned before in the article, women tend to display unusual presentations more than men, which makes them more likely to be disregarded by healthcare professionals, resulting in poor and delayed diagnosis and early discharge of female patients ​[66,67]​. Thus, it is important to impart awareness of the various discrepancies and the combined effect of non-modifiable factors that play a role in CVD.

Some races may have worse outcomes from a CAD; for instance, Black females display a larger number of diseased vessels in contrast to White females when undertaking a first-time CABG, which causes them to remain longer in the ICU and have prolonged hospital stays overall [62]. Race might also affect access to several different and critical treatments; for example, White patients have a higher probability of undertaking PCI and coronary angiography compared to Hispanic patients and patients of the Black race [63].

Ethnicity has also been established as a crucial factor that causes heterogeneity in CAD. A study showed that following the initial coronary angiography, a larger percentage of Asians residing in the USA, more specifically 60% of Asians, were found to have MI in comparison to NHWs [72]. Additionally, classic risk factors such as smoking, diabetes, and obesity also differ among men, women, and subgroups within different ethnicities, races, and ages. Diabetes occurs more often in Hispanics in comparison to NHWs; younger males smoke and drink more alcohol than females, which can be mainly attributed to an unhealthy lifestyle, which subsequently leads to more cases of patients diagnosed with CAD at a younger age [68].

Not only are there heterogeneities in the diagnosis process among individuals with different identities, but treatment management also differs. Specifically talking about age, patients older than 75 years old with CAD tend not to obtain the same amounts of aspirin, ACE inhibitors, and beta blockers in comparison to subjects under 65 years of age [16]. In terms of ethnicity, it has been proven that it accounts for differences in treatment [72]. A study shows that the drug clopidogrel is more frequently prescribed to Asians, in particular 86.2% to Chinese, 83.0% to Filipinos, and 91.4% to Japanese individuals, in contrast to 74.5% of NHWs [72].

As previously mentioned, these factors together contribute to a difference in presentation, diagnosis, and outcomes and are also affected by other risk factors. There are many differences in CAD presentation among individuals who don’t have the same social position; this can cause clinicians to reach different and delayed diagnoses [66,67]. Therefore, healthcare professionals should have a wide range of knowledge on the presentation of CAD amongst different age groups, sexes, and races to administer the best possible care to all patients, and treatment plans can be tailored and optimized to each patient’s needs. This should aid in reducing the mortality rate, attenuating inequalities, and obtaining better outcomes.

Implications for health policy and interventions

The information in the above sections has demonstrated that there are remarkable disparities in CAD presentation, diagnosis, treatment management, and outcomes among individuals of different genders, ethnicities, races, and age groups. Therefore, suggested below is the course of action to diminish these inequalities. 

More work needs to be done to ameliorate doctor-patient relations, break communication barriers, and improve healthcare resource distribution [62]. This might help remove disparities. Public health providers should focus on preventing CAD by targeting modifiable risk factors like diet, exercise, and stress levels [62]. For instance, programs and campaigns are one way to encourage healthy life habits, such as an increase in physical activity and a nutritious diet. For example, offering weekly group workout classes in local community centers for people of all races, ethnic groups, and different age groups. Not only would this allow them to exercise more, but it would also promote socialization, which could alleviate depression and stress. This could be one of the incentives to reduce obesity in society.

One way to improve diet is to set up farmers markets in the community so people can have access to nutritious food. Another way to perhaps control obesity is by providing specialized lunch programs at schools and community centers. Ensuring that these meals are balanced and nutritious could make a big difference and diminish the rate of obesity at a young age. Another measure to promote a better lifestyle would be to prohibit smoking in public places and raise the price of cigarettes. This could perhaps reduce the smoking rate predominantly in male patients since this risk factor is more prevalent in the male sex [9].

Chest pain is a principal manifestation recognized and evaluated by healthcare professionals [66]. So atypical symptoms may not be easily identified and evaluated, making them more susceptible to being disregarded [68]. Therefore, refresher courses should be provided by employers, maybe once a year, so there is a discussion of CAD presentations and treatment plans. Employees must be educated about them in order not to miss the signs of CAD and provide an earlier diagnosis.

Many studies highlight the fact that many patients did not recognize CAD signs and identified those as other symptoms, resulting in them not seeking clinical aid [66]. Thus, it is recommended that there be an increase in routine screening, especially for those with a family history of CAD. Clinicians can also arrange seminars for the public, in particular younger people, to learn about the disease and prevention methods [69].

Other patients, such as Black and Hispanics, despite being aware of their condition, do not undergo the same diagnostic testing or the same treatment plan as those of White patients [63]. Although each patient’s needs are unique, everyone should have the option and opportunity to get the same diagnostic tests and treatments. So, these diagnostic tests and treatments should be offered to all individuals, regardless of their race, ethnicity, age, or sex.

Unfortunately, discrimination and favoritism are still present in many workplaces, including hospitals; thus, it is recommended that employers hire a diverse group of healthcare providers, including clinicians from minority communities. This could potentially enable marginalized Black and Hispanic communities to feel more confident in accessing a hospital or clinic and not feel out of place when seeking medical help and receiving treatments. Consequently, these patients would be able to get vital medical examinations and preventative care [63].

In addition to all of the above recommendations, it is essential to create campaigns to raise awareness regarding the disparities in quality care between insured and uninsured patients, as well as those with higher incomes and educational levels. This could generate a great impact and make politicians and government bodies create better and more accessible healthcare insurance plans and create an outreach program to rural areas where individuals tend to have less opportunity to get an education. Furthermore, governments could provide more funds and scholarships to underprivileged people so they can get better jobs and afford healthcare insurance plans to access the care they need, since the increased quality of medical care frequently indicates a country's economic development [64]. 

Recommendations for future research 

When patients of both sexes diagnosed with MI manifest atypical symptoms, it is vital to consider other factors, such as age, since females have an advanced age when they demonstrate signs of ACS compared to men [66]. So, research with detailed analysis of typical and atypical symptoms related to MI should be conducted and patients of different ages should be included.

Sex is one of the factors that affect CAD. There are great discrepancies between the two genders when it comes to the diagnosis and treatment of AMI [67]. It is recommended that more cross-sectional observational studies be made to further analyze the differences between females and males and to understand how the risk factors and symptoms vary among them, particularly in women since CAD occurrence has been increasing in females. The study should also include large sample sizes to obtain sufficient data for both sexes. Also, females and males should be in the same age group. That way, the only variable would be sex, so the differences found wouldn’t be due to another lurking variable.

Coronary artery disease has been associated with older patients, but in recent years, this condition has been observed increasingly in younger (under 45 years) patients, in particular young females [67]. This might be due to the increased incidence of classic modifiable risk factors such as hypertension, diabetes, and obesity in young women. So far, STEMI and SVD are the patterns of CAD manifestation seen more often in patients under 45 years of age; nevertheless, there isn’t enough data and studies that validate this information, so more research and studies, such as retrospective analysis and multicenter studies, are required. Also, these studies should have a large sample size from a large geographical area. Research studies should take into consideration that the population of a particular geographical area may not necessarily be of the same ethnicity, and it's recommended that specific ethnic groups be selected for studies that aim to evaluate the disparities among them [66].

It is important to continue research on the impact that the intersectionality of sex, age, and race has on CAD. This would benefit epidemiology and public health by expanding comprehension of the relationships that cause inequalities within and between social positions [74]. Additionally, it will provide more data to support the existing studies. The summary of the findings from the referenced studies on the impact of the intersectionality of the three modifiable risk factors (sex, age, and ethnicity) on CAD is in Table 4.

**Table 4:** Overview of the influence of the intersection of sex, ethnicity, and age on the development of CAD CAD: Coronary artery disease, SBP: Systolic blood pressure, CHD: Coronary heart disease, STEMI: ST-elevation myocardial infarction, CABG: Coronary artery bypass grafting, NSTEMI: Non-ST-elevation myocardial infarction, PCI: Percutaneous coronary intervention, IHD: Ischemic heart disease, MI: Myocardial infarction, AMI: Acute myocardial infarction, LAD: Left anterior descending, TVD: Triple-vessel disease, TVR: Target vessel revascularization, ST: Stent thrombosis, NHW: Non-Hispanic White

Identification and Year of Publication	Outcome Measures	Key Findings	Limitations
Pencina et al. (2019) [4]	The focus of this cohort is the comparison of the connection between risk determinants that can be changed, particularly diabetes mellitus, systolic blood pressure (SBP), smoking, lipids, and CHD phenomena, and how the measurements change when adjusted by age and risk factors.	Most of the prognostic data used in the 10-year CHD risk models is predominantly non-modifiable determinants like race, sex, and age.	Some of the information dates back to the 1990s, so it may not represent the changes that happened more recently.
Khera et al. (2015) [61]	The observational study measured temporal patterns and sex-based discrepancies in revascularization strategies and death rates in hospitals for patients with STEMI under 65 years of age.	STEMI is a common CAD presentation in young adults. In contrast with males, it is more improbable for females to receive treatment, such as percutaneous coronary intervention, and they have an elevated death rate in the hospital.	The data may not be representative of more recent trends in management and results for patients under the age of 60 diagnosed with STEMI.
Efird et al. (2015) [62]	The observational study measured the relation between sex and race and the number of vessels affected after a coronary artery bypass grafting procedure. It also measured the end results after the surgery.	Black women CABG patients exhibited a pronounced number of affected vessels in contrast to White women CABG patients. This pattern was not seen among Black and White men. In comparison to other races and sexes, Black females remained for a prolonged period of time in the critical care unit.	This analysis disregarded lesion complexity based on pre-treatment angiographic parameters. The race was self-reported, while social factors information was not collected and taken into account.
Tertulien et al. (2022) [63]	The observational study evaluated individuals presenting with non-ST-elevation myocardial infarction (NSTEMI) and measured the rates of coronary angiography and percutaneous coronary intervention (PCI) by race, ethnicity, and income categories.	Black individuals and Hispanics exhibited lower chances of undergoing PCI and coronary angiography compared to White patients. The lower annual household income also impacted the chances of Asian, Black, and Hispanic individuals to undergo the above procedures.	The data represents only private insured patients, so it cannot be generalized to uninsured patients. Demographic and administrative data are subject to misclassification.
Nowbar et al. (2019) [64]	The observational study measured age-standardized mortality rates and crude death rates for ischemic heart disease (IHD) and other noncommunicable diseases in different countries in which income varied. Sex-based differences were also examined.	The rate of IHD mortality is gradually decreasing in all countries, regardless of population income. The death rate rose as patients got older, and males had a greater mortality rate than females in all the countries selected for this study.	Insufficient information regarding income for some developing countries, particularly Africa, and countries with higher incomes, such as China, is inaccessible. Not all the risk factors were encompassed; information for hypercholesterolemia was left out of this study.
Canto et al. (2012) [65]	The retrospective study analyzed the proportion of individuals hospitalized with myocardial infarction who did not manifest chest pain, the in-hospital death rate, and the relation of age and mortality rate with those lacking chest pain, identifying differences by age, sex, and symptom presentation.	Chest pain was more pronounced in younger (below 65) patients. The mortality rate for patients older than 65 years old was considerably higher than for younger patients. Females did not have chest pain as often as males, and compared to males of the same age, the death rate was more elevated in females.	Patients from NMRI and hospitals that participated might not represent the entire population diagnosed with MI and hospitals in the USA. The definition of MI at the time the study was conducted (< 2007) is different from the actual definition.
George et al. (2021) [66]	The cross-sectional study tried to identify discrepancies in risk determinants, clinical presentation, and coronary angiography among patients of both sexes with MI.	It is more probable for females to show less typical signs and still less probable for them not to go through coronary angiography in contrast to men.	Generalization of discoveries to other populations might be restricted because the research was made in only one tertiary care center in South India. The causality could not be established.
Bajaj et al. (2016) [67]	The observational study focused on assessing the sex discrepancies in clinical manifestation, risk factors, and angiographic disease in patients with acute MI from North India.	Females demonstrate signs of cardiovascular disease when they are older and have an elevated incidence of diabetes and hypertension. While males have a greater prevalence of dyslipidemia, smoking, and BMI, it is more probable for women to have atypical symptoms and insignificant CAD compared to men.	The small sample size prevented them from acquiring enough data on men and women to identify a significant difference. There is a need for age matching between the two groups.
Romero et al. (2013) [68]	The retrospective analysis examined the management of therapeutic procedures and the end-point results for underrepresented females, specifically Hispanics, diagnosed with acute myocardial infarction (AMI) in a hospital in which employees are mainly Hispanics.	Males were often diagnosed with AMI and STEMI and received more treatment than females. Hispanics have less access to private insurance compared to NHW and have less access to different treatments for CAD. Patients with private insurance had a decreased death rate in contrast with those without private insurance.	The findings cannot be applied to the entire Hispanic population since the sample size was not large enough. The large number of Hispanic patients and healthcare providers in the analysis may not accurately represent conditions in other hospitals across the country.
Jariwala et al. (2022) [69]	The observational study explored the incidence and pattern of risk determinants of obstructive CAD in women from India aged below 45 years old in contrast to males with an equal age range who have undergone PCI.	Men are more frequently diagnosed with CAD, but women under 45 can also be affected. ACS is the most frequent pattern observed. TVD is mostly associated with men, while SVD is associated with women. Obesity, dyslipidemia, alcoholism, and smoking have a higher incidence in young males, but classic risk determinants are equally prevalent in young females. Cardiogenic shock rates are similar, and in-hospital mortality is low for both sexes.	The study focused only on three states, not the whole of India. Psychological and socio-economic factors were not considered for the study. The study did not dwell on the details of SYNTAX scores, the number of stents placed, or the procedural complications of PCI.
Hasan et al. (2011) [70]	The retrospective observational study evaluated the differences between White people and South Asians in vessel size and angiographic coronary artery disease severity who are undergoing cardiac catheterization.	South Asians exhibited smaller normalized proximal LAD luminal diameters and more severe CAD compared to White patients; TVD was also more pronounced in South Asians. South Asians may have an increased risk of CAD development and mortality when compared to White patients.	This study could not establish causality or control for all potential confounding variables. The sample size was relatively small. Additionally, not including other ethnic groups could have restricted the ability to compare the findings to those of other populations.
Iantorno et al. (2019) [71]	The retrospective cohort analysis assessed the incidence of major cardiovascular incidents, mortality, myocardial infarction, target vessel revascularization (TVR), and stent thrombosis (ST) within 30 and 365 days post-PCI procedure, remarking potential racial disparities.	In comparison to their White peers, Black females show a more elevated incidence of risk determinants of CAD and show these at a younger age. The TVR and ST were greater in Black females.	Only two ethnicities were taken into account. It did not consider other minorities; hence, it overlooks a major chunk of the population, especially in areas where other minorities are concentrated.
Manjunath et al. (2020) [72]	The observational study aimed to explore different ethnic and racial groups and compare the differences in outcomes and treatment.	Excluding Filipinos, all Asian subgroups received coronary angiography less often than NHW. Diagnostics of MI within one year were more probable among Chinese and Asian Indians, in contrast with NHW. CAD risk is high in Asian Indians and Filipinos, whereas Chinese patients demonstrated a lower risk when compared to NHW. Chinese, Filipinos, and Japanese were prescribed more often clopidogrel than NHW after stenting. More than half of Asians are found to have MI in comparison to NHW.	Small sample size of specific subgroups. Data is subject to coding errors, unmeasured lifestyles, and social factors, which may have an impact on the results.
Li et al. (2016) [73]	The cohort study measured the differences in death among different sexes, racial and ethnic groups, and geographic areas with a duration of 3 years.	On the contrary, females are more likely to get the most effective care during hospital release. Female sex and Black race are associated with higher mortality rates.	The study did not investigate lifestyle and social factors after MI. All patients have insurance. Therefore, the study was not generalized to uninsured patients. Patients self-declared their ethnic and racial groups. The sample size was composed of individuals who were 65 years of age and older.

## Conclusions

Coronary artery disease is a multifactorial condition and is one of the leading causes of death in the world. Demographic factors greatly influence this disease and need to be taken into consideration when evaluating a patient since there are many heterogeneities among patients of different ethnicities, races, sexes, and ages. Some patients are more affected than others; those with an advanced age, female sex, Black race, and Hispanic ethnicity have a higher risk of having CAD. The severity of CAD and the mortality rate are greater for these patients. Not having a comprehensive health insurance plan and a higher household income in combination with a lack of collegiate education also contribute to the disparities within the population by lowering the chances of them getting appropriate treatment.

Despite the studies made in the last 15 years emphasizing many of the differences, there is still a big gap that needs to be filled. So, it is vital to continue studying the influence and intersectionality of sex, age, race, and ethnicity on the risk, presentation, and outcomes of CAD to impart awareness and make healthcare professionals comprehend how these factors cause disparities in the population. Knowing that certain risk factors, symptoms, outcomes, and responses to treatments are more prevalent in certain social categories will make it possible to provide better quality care. The results of this change can range from earlier diagnoses of CAD to a quicker and more personalized treatment plan for each patient’s specific needs, thereby decreasing morbidity and mortality and improving quality of life.
